# Permanent and Regenerative Grazing Systems Are Associated with Distinct Fecal Bacterial and Fungal Microbiota Patterns in Donkeys Under Extensive Pasture Conditions

**DOI:** 10.3390/ani16203288

**Published:** 2026-10-19

**Authors:** Ákos Juhász, Julianna Katalin Bokor, Norbert Boros, Sándor Csete, Réka Erika Döbröntey, Márton Herényi, András Ittzés, Lola Virág Kiss, János Nagy, Péter István Nagy, Ferenc Olasz, Marianna Oláh, Miklós Sárospataki, Anikó Seres, Katalin Posta

**Affiliations:** 1 Agribiotechnology and Precision Breeding for Food Security National Laboratory, Institute of Genetics and Biotechnology, Hungarian University of Agriculture and Life Sciences, 2100 Gödöllő, Hungary; olasz.ferenc.gyorgy@uni-mate.hu (F.O.); posta.katalin@uni-mate.hu (K.P.); 2 MATE Practical Farm Nonprofit Ltd., Game Management Centre, 7475 Bőszénfa, Hungary; bokor.julianna.katalin@uni-mate.hu (J.K.B.); nagy.janos@uni-mate.hu (J.N.); 3 Department of Soil Science, Institute of Environmental Sciences, Hungarian University of Agriculture and Life Sciences, 2100 Gödöllő, Hungary; boros.norbert@uni-mate.hu (N.B.); dobrontey.reka.erika@uni-mate.hu (R.E.D.); 4 Department of Nature Conservation Biology, Institute for Wildlife Management and Nature Conservation, Hungarian University of Agriculture and Life Sciences, 7400 Kaposvár, Hungary; csete.sandor@uni-mate.hu; 5 Department of Zoology and Ecology, Institute for Wildlife Management and Nature Conservation, Hungarian University of Agriculture and Life Sciences, 2100 Gödöllő, Hungary; herenyi.marton.farkas@uni-mate.hu (M.H.); kiss.lola.virag@uni-mate.hu (L.V.K.); nagy.peter.istvan@uni-mate.hu (P.I.N.); sarospataki.miklos@uni-mate.hu (M.S.); seres.aniko@uni-mate.hu (A.S.); 6 Department of Applied Statistics, Institute of Mathematics and Basic Science, Hungarian University of Agriculture and Life Sciences, 1118 Budapest, Hungary; ittzes.andras@uni-mate.hu; 7 Research Center for Agro-Environmental Sciences, Institute of Environmental Sciences, Hungarian University of Agriculture and Life Sciences, 2100 Gödöllő, Hungary; olah.marianna@uni-mate.hu

**Keywords:** donkey, grazing management, rotational grazing, amplicon sequencing, fecal microbiota, bacterial communities, fungal communities, microbial diversity

## Abstract

This study investigated whether permanent and regenerative (rotational) grazing systems are associated with differences in fecal bacterial and fungal microbiota of donkeys under extensive pasture conditions. Young female donkeys were assigned to permanent or regenerative grazing, and paired fecal samples from eight animals per system were collected before pasture access and at the end of the grazing period. Culture-based microbial counts, parasitological examination, animal performance measurements, forage assessment, and bacterial 16S rRNA gene and fungal ITS amplicon sequencing were performed. Donkeys under regenerative grazing showed higher average daily gain than those under permanent grazing (378.0 ± 57.8 vs. 242.4 ± 65.8 g/day; *p* < 0.001), whereas culture-based fecal microbial counts and fecal egg counts did not differ significantly between systems. Beta-diversity analyses identified sampling time as a consistent source of variation in both bacterial and fungal communities, whereas associations with grazing system and its interaction with sampling time depended on the distance metric used. Bacterial communities retained a large shared core, whereas fungal communities showed stronger compositional turnover. Longitudinal differential abundance analysis identified 336 bacterial and 66 fungal amplicon sequence variants (ASVs) with significant grazing system × sampling time interactions. More positive temporal responses under regenerative grazing were observed among several Spirochaetota and Neocallimastigomycota ASVs, whereas detected *Methanobrevibacter* ASVs showed a more positive temporal change under permanent grazing.

## Simple Summary

Grazing management can influence not only pasture condition and animal performance, but also the microorganisms living in the digestive tract of grazing animals. Donkeys are important animals in extensive and low-input grazing systems, yet little is known about how their fecal bacterial and fungal communities respond to different pasture management practices. In this study, young female donkeys were kept either under permanent grazing, where animals had continuous access to the same pasture, or under regenerative grazing, where animals were moved between paddocks to allow vegetation regrowth. Fecal samples were collected before pasture access and at the end of the grazing period. Donkeys under regenerative grazing showed higher weight gain. Conventional culture-based microbial counts did not differ between grazing systems, but DNA-based microbiome analysis revealed changes in bacterial and fungal community structure associated with sampling time and grazing system. Bacterial communities were largely shared among groups, whereas fungal communities showed stronger changes over time and between grazing systems. These results suggest that contrasting pasture-management contexts can be associated with measurable differences in the fecal microbiota of donkeys and highlight the value of including fungal communities in studies of herbivore digestive ecology.

## 1. Introduction

Grassland ecosystems cover a substantial proportion of the terrestrial surface of Earth and play a key role in global food production, carbon cycling, and biodiversity conservation. Grazing by large herbivores is one of the most important ecological drivers shaping grassland structure, plant species composition, and ecosystem functioning [1,2]. Through selective feeding, trampling, and nutrient return via dung and urine, grazing animals influence vegetation dynamics, soil physical and chemical properties, and belowground biological communities [3,4].

In recent decades, climate change has increasingly challenged the viability of conventional grazing practices. Rising temperatures, altered precipitation regimes, and more frequent drought events have intensified pressure on grassland ecosystems, particularly under continuous or poorly managed permanent grazing systems [5,6]. Under such conditions, permanent grazing may exacerbate overgrazing, reduce plant cover and diversity, and impair soil structure and biological activity, ultimately decreasing system resilience [7,8]. These challenges have stimulated growing interest in alternative grazing strategies designed to maintain productivity while supporting ecological functioning.

Regenerative and rotational grazing systems have been proposed as management approaches that better align livestock production with ecological processes. Although definitions vary, regenerative grazing generally emphasizes controlled stocking density, planned rest periods for vegetation recovery, and the enhancement of soil biological activity and organic matter content [9,10]. Importantly, the term “regenerative grazing” is used inconsistently in the literature, often encompassing management systems with differing ecological outcomes, which complicates cross-study comparisons and interpretation of reported effects [11,12].

Several studies have reported that adaptive multi-paddock or rotational grazing can improve plant community composition, increase soil carbon stocks, and enhance soil microbial biomass and activity compared to continuous grazing [13,14,15]. Nevertheless, the magnitude and consistency of these effects remain debated, particularly across climatic regions, reflecting the strong context dependency of grazing management outcomes [16,17]. Empirical evidence from adaptive multi-paddock grazing systems suggests that improvements in soil carbon stocks and biological indicators are strongly influenced by local environmental conditions and prior land use, underscoring the need for farm-scale comparisons [18]. Consequently, grazing systems that systematically modify plant community structure and forage quality, such as regenerative grazing, may exert cascading effects on herbivore nutrition and gut microbial communities.

Grazing-induced changes in vegetation composition and forage quality have direct consequences for the gastrointestinal microbiota of herbivorous animals. The gut microbiota plays a central role in nutrient utilization, host health, and metabolic efficiency, particularly in herbivores relying on microbial fermentation to degrade plant structural carbohydrates [19,20]. Diet is considered one of the strongest determinants of gut microbial community composition, and shifts in forage type, maturity, and chemical composition can rapidly alter microbial structure and function [21,22]. Therefore, grazing management, by shaping plant communities and forage availability, represents an indirect but powerful driver of herbivore gut microbiota.

The donkey hindgut is highly developed and comprises the cecum, colon, and rectum, forming a complex microbial ecosystem inhabited by dense and diverse communities of bacteria, anaerobic fungi, methanogenic archaea, and protozoa [23,24]. Donkeys rely on this resident hindgut microbiota to degrade dietary plant fiber and meet their energy requirements, making microbial fermentation central to their nutrition and physiological performance [25,26]. Multi-kingdom analyses have demonstrated that the donkey hindgut microbiome comprises interacting bacterial, archaeal, and fungal communities whose composition varies along the gastrointestinal tract and differs between intestinal contents and feces [27].

Available studies on donkey gut microbiota have so far focused primarily on bacterial communities, mostly using 16S rRNA gene amplicon sequencing under controlled or semi-controlled feeding conditions [28,29]. These studies have provided valuable baseline information on bacterial community composition and its relationship to diet and digestive efficiency. Studies employing high-throughput sequencing and multi-omics approaches have further demonstrated that diet, physiological status, and feeding strategy are major determinants of donkey gut microbial structure and predicted metabolic function [30,31,32]. In contrast, only very limited work has included fungal communities in donkey gut microbiome analyses [28]. Comprehensive ITS-based characterization of donkey-associated fungal communities also remains scarce, particularly under grazing conditions.

Most research on grazing management and gut microbiota has focused on ruminant livestock, particularly cattle and sheep, reflecting their dominant role in global meat and milk production [21,33]. In contrast, comparatively little attention has been paid to hindgut fermenters such as equids, despite their widespread use in extensive and semi-natural grazing systems. Donkeys (*Equus africanus asinus* Linnaeus) play an important ecological and socio-economic role in low-input and marginal environments, where they are commonly integrated into extensive grazing systems and often coexist with ruminants on shared pastures [34]. As monogastric herbivores relying on hindgut fermentation, donkeys differ fundamentally from ruminants in digestive physiology, feeding behavior, and nutrient utilization, which may result in distinct interactions between grazing management, diet, and gut microbiota.

Although equine gut microbiota has been shown to respond strongly to dietary composition and feeding management, most available studies have been conducted under controlled feeding conditions or have focused primarily on horses rather than donkeys [35,36]. Data on donkey gut microbiota under extensive grazing systems remain limited, particularly with respect to grazing management effects. Recent comparative studies suggest that donkey gut microbial communities exhibit distinct structural and functional features compared to other equids and show marked sensitivity to feeding strategy and physiological state [37,38]. The limited knowledge of donkey gut microbiota under grazing conditions is especially relevant for anaerobic fungi, which play a crucial role in the degradation of lignocellulosic plant material in herbivore digestive tracts through their potent fiber-degrading enzyme systems and physical penetration of plant tissues [39,40]. Despite their recognized functional importance, fungal communities are rarely included in microbiome studies of non-ruminant herbivores, including equids [28,40,41,42]. Recent donkey-focused studies indicate that anaerobic gut-associated fungi constitute a substantial and dynamic component of the hindgut ecosystem and may respond rapidly to dietary composition and feeding management, underscoring the need for their inclusion in grazing-related microbiome studies [27,38].

From an ecosystem perspective, the presence of both ruminant and monogastric herbivores on the same pasture may further increase system complexity. Different digestive strategies can lead to differences in forage selection, digestion efficiency, and microbial outputs returned to the environment via feces, potentially influencing nutrient cycling and microbial feedbacks at the soil–plant interface [43,44,45,46]. Recent evidence suggests that grazing animals may also act as vectors linking soil, plant, and gut microbial communities, reinforcing feedbacks between pasture management and host-associated microbiomes [47].

Despite growing interest in regenerative grazing and increasing recognition of the importance of gut microbiota for animal and ecosystem health, studies integrating grazing management with comprehensive microbiome analyses in donkeys remain extremely limited. To our knowledge, no previous studies have simultaneously examined bacterial and fungal fecal communities of donkeys under contrasting grazing systems in extensive, mixed-species pasture environments. Therefore, the objective of the present study was to compare the fecal microbiota of donkeys managed under permanent and regenerative grazing systems, with a focus on both bacterial and fungal community composition and associated animal performance under field conditions. We hypothesized that regenerative grazing, by promoting planned rest periods, forage regrowth, and more stable forage availability, would be associated with detectable shifts in fecal microbial community structure and the relative abundance of fiber-associated microbial taxa compared to permanent grazing. By including both bacterial and fungal communities in an underrepresented host species, this study provides a field-based perspective on the links between grazing management, forage conditions, animal performance, and fecal microbiota in extensive grassland systems.

## 2. Materials and Methods

### 2.1. Study Site and Grazing Areas

The study was carried out at the Bőszénfa Deer Farm (Hungary), a 1336-ha agricultural area operated by MATE Practical Farm Nonprofit Ltd. (Bőszénfa, Hungary), an educational and research farm affiliated with the Hungarian University of Agriculture and Life Sciences (MATE). The farm maintains several livestock species, including donkeys, water buffalo, horses, and multiple deer species, under extensive grazing systems.

Two pasture areas with comparable size and general vegetation characteristics were selected for the study. One area (5.3 hectares) was managed under a permanent (continuous) grazing system, where animals had unrestricted access to the entire pasture throughout the grazing period. The second area (5.8 hectares) was managed under a regenerative (rotational) grazing system and was divided into multiple paddocks using electric fencing. Each management context was represented by a single pasture. Accordingly, pasture identity and grazing management were not independently replicated and cannot be separated experimentally in the present field study. The study should therefore be interpreted as an exploratory field comparison of two pasture-management contexts rather than as a replicated grazing-system experiment. For clarity, the terms “permanent grazing system” and “regenerative grazing system” are retained throughout the manuscript as operational labels for these two specific pasture-management contexts.

### 2.2. Study Design, Grazing Management, and Study Timeline

Hungarian native donkeys (*Equus africanus asinus* Linnaeus, Hungarian breed name: “magyar parlagi szamár”) and Hungarian domestic water buffalo (*Bubalus bubalis* Linnaeus, Hungarian breed name: “magyar házi bivaly”) were included in the grazing system as part of the farm’s routine livestock population. Only female yearling animals were included in the study.

Prior to grazing, animals were housed in a stable and received the same grass hay diet (see Table S1 for feed composition) from the beginning of December 2024 until the start of the grazing period (five months). At the start of the grazing period, the donkeys were 11 ± 1 months of age. Individual body weight measurements and baseline fecal sampling were performed on 24 April 2025, prior to pasture access. Final body weight measurements were performed on 15 July 2025, resulting in an 82-day interval between the two body weight measurements, of which the animals spent 70 days on pasture. At the time of baseline sampling, animals were still housed in the stable and had not yet been exposed to pasture vegetation. As part of routine herd management, animals within each species were randomly allocated to two grazing groups (donkeys: *n* = 12 per group; water buffaloes: *n* = 10 per group) using random numbers generated with the RANUNI function and a fixed seed in SAS OnDemand for Academics (SAS Institute Inc., Cary, NC, USA). Baseline body weight balance was subsequently evaluated using Student’s *t*-test (PROC TTEST), and no significant differences in initial body weight were detected between the two groups in either species (*p* > 0.05). Individual animals constituted the animal-level observational units for repeated measurements within each pasture-management context and did not represent independent replicates of the pasture-level grazing management.

Animals were moved to their respective pasture areas on 7 May 2025. Grazing management in both pasture areas was part of routine farm management and involved mixed-species grazing, with donkeys and water buffaloes grazing the same pasture areas throughout the study period. The present study focused exclusively on donkeys, as only donkey fecal samples were collected and processed in a standardized manner suitable for culture-based microbiological analyses and high-throughput amplicon sequencing. Animals assigned to the continuous (permanent) grazing system had unrestricted access to the entire pasture area. Animals assigned to the regenerative grazing system, implemented through rotational paddock use, were managed on a pasture subdivided into 21 paddocks (with an average size of 2768 m^2^) using electric fencing and were moved to a new paddock every two days according to a predefined grazing schedule. This schedule was designed to minimize repeated grazing of newly regrowing vegetation and to provide approximately 40 days or more of recovery between grazing events. The term regenerative grazing is therefore used here to indicate that rotational livestock movement was combined with planned vegetation recovery as an explicit management objective, rather than referring to rotation alone. Stocking density was comparable between grazing systems and ranged from 1.46 to 1.60 livestock units per hectare at the start of the grazing period and from 1.79 to 1.88 livestock units per hectare at the end of the grazing period (Table S2). One livestock unit corresponded to 500 kg of live weight. Mineral salt blocks (Na: 38%, Ca: 0.5%, Mg: 0.1%, Zn: 1000 mg/kg, Mn: 1000 mg/kg, Fe: 200 mg/kg, I: 100 mg/kg, Se: 20 mg/kg) were provided in both grazing systems, and drinking water was supplied *ad libitum* using water troughs.

The original study design aimed to maintain grazing until autumn. However, due to severe drought conditions during late spring and summer 2025, pasture regrowth was strongly limited. To ensure comparable management conditions, animals were removed from both grazing systems simultaneously, and the study was terminated on 15 July 2025, resulting in a total grazing period of 70 days.

Meteorological data, including daily temperature and precipitation, were obtained from an on-site automated Boreas meteorological station located within the permanently grazed pasture area and were used to characterize weather conditions during the grazing period (Figure S1). Long-term monthly reference values for the 1991–2021 climatological normal period were obtained from HungaroMet Hungarian Meteorological Service and used for comparison with the study period.

### 2.3. Feed and Pasture Vegetation Assessment

The chemical composition of the grass hay provided during the pre-grazing stable period was determined according to standard Hungarian feed analytical methods at the Central Analytical Laboratory of the Hungarian University of Agriculture and Life Sciences, Kaposvár, Hungary. Measured parameters included moisture, crude protein, crude fat, crude fiber, crude ash, and structural fiber fractions, including neutral detergent fiber (NDF), acid detergent fiber (ADF), and acid detergent lignin (ADL). Except for moisture content, nutrient concentrations are expressed as g/kg on a dry matter (DM) basis (Table S1).

Vegetation sampling was conducted on 4 May and 18 June. Samples from 4 May were collected shortly before the animals were released onto the pasture, while the samples from 18 June were taken one month prior to the time the animals were removed from the pasture. On 4 May, plant biomass samples were collected in the same manner on both pastures at six random sampling points. Two sub-samples were collected at each point, spaced 25 m apart. During the collection of the sub-samples, plant biomass was removed from a 10 cm × 250 cm strip, corresponding to a total area of 2500 cm^2^ (0.25 m^2^), using handheld grass shears (Bosch AdvancedShear 18V-10, Robert Bosch Power Tools GmbH, Leinfelden-Echterdingen, Germany). The tool was slid along the soil surface at a height of approximately 1 cm. The grass samples were then bagged and shipped the same day for laboratory analysis.

After weighing the two sub-samples and determining their moisture content, they were mixed together, and the detailed compositional characteristics were determined from the resulting composite sample. Laboratory analyses were conducted in the same laboratory and using the same methods as for the pre-grazing grass hay samples. For pasture vegetation samples, the following parameters were determined: moisture content, crude protein, crude fat, crude fiber, crude ash, NDF, ADF, ADL, hemicellulose, and cellulose. Except for moisture content, nutrient concentrations are reported as g/kg DM.

During the 18 June sampling, the above-mentioned method was used again on the permanent pasture at the same six random sampling points used in May. In this case, the sub-samples were taken 4 m north and south of each of the previously used sampling locations. On the regenerative pasture, a different approach was used in June. Since the animals had already grazed nearly all of the 21 paddocks by that time, except for the last two paddocks, random samples were collected from the first three paddocks where new vegetation had regrown for 42 days after grazing. Two sub-samples were collected from each paddock. Accordingly, the June sampling comprised six spatial sampling units in the permanent pasture and three regrown paddocks in the regenerative pasture, avoiding the inclusion of paddocks representing different stages of grazing or regrowth.

### 2.4. Animal Measurements and Fecal Sampling

Individual body weight measurements and fecal sample collection were performed concurrently at two time points: before the start of grazing (24 April 2025) and at the termination of the grazing period (15 July 2025). Procedures were carried out as part of routine herd management using standard farm equipment. Body weight was measured individually using a calibrated livestock scale (METRISOFT, Hódmezővásárhely, Hungary; model MS-HD10/KN-ALL).

Fecal samples were collected non-invasively during routine handling. Fresh fecal material was collected using disposable gloves and was assigned to individual animals based on direct observation at the time of defecation. Samples were placed into sterile containers, transported to the laboratory under cooled conditions, and processed on the day of collection. Following homogenization, aliquots of fecal material were prepared for culture-based analyses, while a subsample (150 mg) from each sample was stored at −80 °C for subsequent DNA extraction and amplicon sequencing. Although fecal sampling was attempted for all animals at both sampling time points, complete paired fecal samples were not available for every individual. Therefore, only animals with fecal samples successfully collected at both time points were included in downstream microbiological and molecular analyses. This resulted in a final dataset of eight donkeys per grazing group for culture-based and amplicon sequencing microbiota analyses and parasitological examination.

Fecal egg counts for parasitological analyses were assessed using a modified McMaster method described earlier [48]. Three grams of fecal samples were homogenized in 42 mL of tap water, and then centrifuged in 15 mL tubes. After centrifugation, the supernatant was discarded, and the sediment was resuspended and adjusted to 45 mL with flotation fluid (zinc sulfate with a specific gravity of 1.37). For egg calculation, both sides of a McMaster counting chamber were filled twice. The number of parasite eggs per gram (EPG) was calculated by multiplying the total number of eggs by a coefficient of 25.

### 2.5. Culture-Based Microbiological Analysis

One gram of homogenized fecal material from each sample was used for culture-based microbiological analyses. Serial dilutions were prepared in sterile physiological saline, and diluted samples were plated in duplicate on selective and non-selective culture media. De Man, Rogosa and Sharpe Agar was used for the enumeration of lactic acid bacteria (LAB), Eosin Methylene Blue Agar for *Enterobacteriaceae*, Nutrient Agar for total aerobic bacteria, and Columbia Blood Agar for total anaerobic bacteria. Total aerobic fungi were enumerated on Rose-Bengal Chloramphenicol Agar. All culture media were purchased from Biolab Inc. (Budapest, Hungary). Bacterial plates were incubated at 37 ± 1 °C for 24–48 h under aerobic or anaerobic conditions, as appropriate for each microbial group. Fungal plates were incubated aerobically at 37 ± 1 °C for 72 h. Colonies were counted on plates containing countable numbers of colonies, and results were expressed as colony-forming units (CFU) per gram of feces (CFU/g, wet weight). CFU values were log10-transformed prior to statistical analysis.

### 2.6. DNA Extraction and Amplicon Sequencing

Genomic DNA was extracted from 150 mg of fecal material obtained from eight individuals per grazing group at each sampling time using the ZymoBIOMICS 96 MagBead DNA Kit (Zymo Research, Irvine, CA, USA), following the manufacturer’s protocol. DNA concentration was quantified using the Quant-iT dsDNA BR Assay Kit (Thermo Fisher Scientific, Waltham, MA, USA) on an Infinite Pro 200 F Nano+ Fluorescent Plate Reader (Tecan, Männedorf, Switzerland).

Bacterial and fungal community composition was characterized by high-throughput amplicon sequencing. The V3 –V4 region of the 16S rRNA gene was amplified using tagged primers described by Klindworth et al. (2013) [49], and library preparation followed the Illumina 16S Metagenomic Sequencing Library Preparation Guide [50]. Although this primer set can also amplify archaeal 16S rRNA sequences, it is primarily used for bacterial community profiling and is not specifically optimized for comprehensive archaeal coverage. Archaeal sequences detected in the dataset were therefore interpreted with consideration of potential primer-related bias. Fungal communities were assessed by amplifying the ITS region using tagged ITS1F and ITS4R primers according to the Illumina 16S and Fungal Metagenomic Sequencing Library Preparation Guides [51]. Briefly, 50 ng of template DNA was used for the initial PCR amplification (PCR1), which was carried out using KAPA HiFi HotStart Ready Mix (Roche AG, Basel, Switzerland) for 25 cycles. Amplicons were purified, and 10 ng of the purified PCR product was used as input for index PCRs with the Nextera XT Index v2 Kit (Illumina, San Diego, CA, USA) and KAPA HiFi HotStart Ready Mix, using eight amplification cycles. Indexed libraries were purified with 0.8× volume KAPA Pure Beads (Roche AG, Basel, Switzerland). Library concentrations were determined using the Equalbit 1× dsDNA HS Assay Kit (Vazyme Biotech, Nanjing, China), and fragment size distributions were assessed by capillary electrophoresis using a LabChip GX Touch HT Nucleic Acid Analyzer and the DNA NGS 3K Assay Kit (Revvity, Waltham, MA, USA). Libraries were normalized to equimolar concentrations, pooled, and sequenced using 2 × 300 bp paired-end chemistry on an Illumina NextSeq 2000 platform at Xenovea Ltd. (Szeged, Hungary).

### 2.7. Bioinformatics Processing and Taxonomic Assignment

Raw sequencing reads were used for downstream bioinformatics analyses. Demultiplexing and removal of sequencing adapters were performed using the instrument’s standard control software. Quality filtering was applied to exclude low-quality reads prior to downstream analyses. Paired-end reads targeting the bacterial 16S rRNA gene and the fungal ITS region were processed using the FROGS pipeline v4.2.0 [52]. Briefly, forward and reverse reads were quality-filtered and merged using VSEARCH v2.17.0 [53] with the following parameters: minimum amplicon length of 150 bp, maximum length of 600 bp, and mismatch rate of 0.1. Merged reads were subsequently clustered into sequence units using the SWARM v3.0.0 algorithm [54]. In the current FROGS workflow (v4.2.0), the resulting post-filtering features are reported as amplicon sequence variants (ASVs). Accordingly, the term ASV is used throughout this study following the current FROGS output terminology and refers here to SWARM-derived amplicon sequence clusters rather than exact sequence variants inferred by denoising algorithms such as DADA2 or Deblur. Chimeric sequences were identified and removed using the chimera detection tools implemented within the FROGS workflow. To reduce noise and potential artifacts, singleton sequences and low-abundance clusters representing less than 0.005% of the total read count were excluded from further analyses. Taxonomic assignment of bacterial 16S rRNA gene sequences was performed using BLAST v2.16.0 [55] against the SILVA 138.2 reference database [56], while fungal ITS sequences were classified against the UNITE Fungi 10 database [57]. The resulting abundance tables and taxonomic annotations were used as input for downstream diversity, community composition, and differential abundance analyses.

### 2.8. Microbiome Data Analysis

Different downstream analyses were performed on either the complete filtered, non-rarefied abundance tables or rarefied abundance tables, depending on the analytical requirements. ASV richness summaries and taxonomic composition analyses were based on the complete filtered, non-rarefied dataset, with relative abundances calculated from these data where appropriate, whereas alpha- and beta-diversity analyses were performed on rarefied abundance tables. For bacterial 16S rRNA datasets, samples were rarefied to 105,064 reads per sample, while fungal ITS datasets were rarefied to 15,284 reads per sample. Rarefaction curves indicated that sequencing depth was sufficient to capture the majority of ASV richness across all groups for both bacterial and fungal communities. No samples were excluded due to low read counts. Alpha and beta diversity metrics were calculated using the phyloseq package (v1.38.0) implemented within the FROGS framework [58]. Alpha diversity was assessed using observed ASV richness, the Chao1 richness estimator, the Shannon diversity index, and the inverse Simpson index. To account for the paired sampling design, changes in alpha diversity were assessed at the individual level by calculating the difference between final and start samples for each animal. These individual-level changes were compared between permanent and regenerative grazing systems using the Mann–Whitney U test. For each microbial domain separately, *p*-values obtained for the four alpha-diversity indices were adjusted for multiple testing using the Benjamini–Hochberg false discovery rate correction, and adjusted *p*-values < 0.05 were considered statistically significant.

Beta diversity of bacterial and fungal communities was assessed using unweighted and weighted UniFrac distance metrics. Ordination of beta diversity distance matrices was performed by principal coordinates analysis (PCoA) to visualize patterns in community structure across grazing systems and sampling times. Differences in community composition were statistically evaluated using permutational multivariate analysis of variance (PERMANOVA) with the adonis2 function implemented in the vegan package (v2.7-3), based on 9999 permutations. Permutation matrices were generated using the permute package (v0.9-10). For testing the between-animal contrast between grazing groups, the model was specified as distance ~ time * grazing, and animals were permuted as whole units between grazing systems while preserving the paired start and final observations within each animal. This was implemented by treating individual animals as freely permutable plots while disabling permutation within animals. For testing the within-animal sampling-time effect and the grazing-system × sampling-time interaction, the model was specified as distance ~ grazing * time, with permutations restricted within individual animals. For these tests, animal identity was used as a blocking factor and permutation was allowed only within animals. Homogeneity of multivariate dispersion was assessed using PERMDISP with the betadisper function in vegan, based on distances to group medians followed by permutation testing. Dispersion was evaluated for grazing system, sampling time, and the four grazing × sampling-time combinations using whole-animal, within-animal, and split-plot permutation schemes, respectively. For the split-plot scheme used for the four grazing × sampling-time combinations, individual animals were treated as freely permutable plots and permutation was also allowed within animals.

Differential abundance analysis was performed separately for bacterial and fungal communities using the DESeq2 package (v1.46.0) [59]. The complete filtered, non-rarefied count matrix was used as input for DESeq2; no rarefaction or relative-abundance transformation was applied prior to the analysis. Size factors were estimated using the poscounts method. To account for the paired longitudinal design and baseline variability between grazing groups, the exact design formula was ~ grazing + time + grazing:ind_n + grazing:time, where ind_n represented individual animals nested within grazing system. Permanent grazing and the start sampling point were used as the reference levels for grazing system and sampling time, respectively. The interaction term tested whether the temporal change in ASV abundance differed between permanent and regenerative grazing systems. Wald tests were used for statistical inference. *p*-values were adjusted for multiple testing using the Benjamini–Hochberg false discovery rate correction, and ASVs with an adjusted *p*-value < 0.05 were considered statistically significant. Positive interaction log_2_ fold-change values indicate a more positive temporal change under regenerative grazing relative to permanent grazing, whereas negative values indicate the opposite pattern. In addition, potential biomarker taxa were identified in R using the LEfSe algorithm as implemented in the microeco package (v2.3.0), which combines non-parametric statistical testing with linear discriminant analysis (LDA) to detect taxa with consistent differences in abundance across groups at multiple taxonomic ranks [60]. LEfSe was performed on taxonomic relative-abundance tables calculated from the complete filtered, non-rarefied count data. The default microeco LEfSe settings were retained, including a significance threshold of *p* < 0.05, false discovery rate adjustment of *p*-values, and abundance normalization to 1,000,000. No additional LDA-score threshold was imposed; the top 30 significant taxa ranked by LDA score were displayed in the corresponding figures.

### 2.9. Additional Statistical Analyses

All other statistical analyses were conducted in R version 4.4.3 [61]. For pasture vegetation data, forage biomass and nutrient composition were compared between the spatial sampling units of the permanent and regenerative pastures separately for each sampling date. Normality of the data was assessed prior to testing. As the data did not deviate from normality, differences between the two sampled pastures were evaluated using two-sample *t*-tests. Results are presented as mean ± standard error, and *p* < 0.05 was considered statistically significant. These comparisons were used to characterize differences between the two specific pastures at each sampling date; the within-pasture spatial sampling units were not considered independent replicates of the pasture-level grazing management. Individual body weight measurements were performed before the start and at the termination of the grazing period. Average daily gain (ADG) was calculated for each animal over the 82-day interval between the two body weight measurements, and differences between grazing systems were assessed using Welch’s *t*-test to account for unequal variances, with effect size estimated by Cohen’s d. Data were visualized using boxplots showing medians, interquartile ranges, and individual values. For culture-based microbial counts, log_10_-transformed CFU values were summarized using descriptive statistics. Changes over time were evaluated at the individual level by calculating differences between final and initial values, which were compared between grazing systems. Venn diagram analyses were performed using the complete filtered, non-rarefied ASV abundance tables, whereas heatmap visualizations were based on relative abundance data, using the VennDiagram (v1.8.2) and ComplexHeatmap (v2.22.0) packages, respectively. Heatmap values were scaled using a row-wise z-score transformation, calculated as z = (x − μ)/σ, where x represents the relative abundance of a given taxon in a sample, and μ and σ denote the mean and standard deviation of that taxon across grazing groups. Across all statistical analyses, comparisons involving grazing systems were interpreted as contrasts between the two observed pasture-management contexts.

### 2.10. Use of Generative Artificial Intelligence

During the preparation of this manuscript, the authors used ChatGPT (OpenAI, GPT-5.5 Thinking) to assist with language editing, improving clarity and readability, and refining the wording of selected manuscript sections based on author-provided content. The authors reviewed and edited all output generated with the assistance of Generative Artificial Intelligence and take full responsibility for the content of this publication.

## 3. Results

### 3.1. Forage Availability and Nutritional Characteristics During the Grazing Period

Before the start of the grazing period, all donkeys were housed under the same stable conditions and received the same grass hay diet for five months. This common pre-grazing feeding period provided a uniform nutritional background before animals were allocated to the two grazing systems. The chemical composition of the grass hay is provided in Table S1.

Weather conditions during the study period were characterized by below-average precipitation compared with the 1991–2021 monthly reference values provided by HungaroMet (Table 1). Precipitation was lower than the long-term monthly average throughout April-July 2025, with the strongest deficit occurring in June, when only 3 mm of precipitation was recorded compared with the 1991–2021 reference value of 85 mm. Although total monthly precipitation in July was 38 mm, only 10 mm fell before the end of the grazing period on 15 July, whereas most of the remaining rainfall occurred after the animals had already been removed from the pastures. Monthly mean temperatures were broadly comparable to the long-term reference values, but the near-total lack of rainfall in June strongly limited pasture regrowth. Daily temperature, precipitation, and relative humidity patterns during the grazing period are shown in Figure S1.

At the beginning of grazing, forage biomass and nutrient composition were similar between the permanent and regenerative pastures (Table 2). On 4 May, dry matter biomass was 134.92 ± 9.11 g/0.25 m^2^ in the permanent pasture and 115.20 ± 14.48 g/0.25 m^2^ in the regenerative pasture. Crude protein content was also similar between pastures at this time (126.2 ± 4.6 g/kg DM and 123.8 ± 3.7 g/kg DM, respectively), as were the main fiber fractions, including NDF (524.4 ± 9.7 g/kg DM and 499.5 ± 29.3 g/kg DM) and ADF (308.8 ± 6.7 g/kg DM and 294.3 ± 19.2 g/kg DM).

By 18 June, forage composition had shifted in both pastures toward a drier and more fibrous state. Moisture content decreased from 78.26% to 48.89% in the permanent pasture and from 79.43% to 58.94% in the regenerative pasture. Crude protein content declined in both pastures, from 126.2 g/kg DM to 69.8 g/kg DM in the permanent pasture and from 123.8 g/kg DM to 85.7 g/kg DM in the regenerative pasture. In parallel, crude fiber increased from 261.8 g/kg DM to 320.0 g/kg DM in the permanent pasture and from 251.1 g/kg DM to 318.4 g/kg DM in the regenerative pasture. Similar increases were observed for NDF, ADF, and cellulose. These changes indicate that the available forage became progressively more mature and fiber-rich during the dry grazing period.

In June, samples collected from the regenerative pasture had significantly higher moisture content than those from the permanent pasture (58.94 ± 2.31% vs. 48.89 ± 1.90%; *p* < 0.05), as well as higher crude fat content (26.2 ± 2.2 g/kg DM vs. 21.5 ± 0.7 g/kg DM; *p* < 0.05). Crude protein also tended to be higher in the regenerative pasture (85.7 ± 11.5 g/kg DM vs. 69.8 ± 2.6 g/kg DM), although this difference was not significant (*p* > 0.05). The vegetation data indicate that both pastures were exposed to substantial seasonal drying, while the regenerative paddocks maintained less desiccated regrowth forage at the June sampling point.

### 3.2. Grazing Performance and General Animal Health

At the animal level, donkeys in the regenerative grazing group had a higher ADG over the 82-day weighing interval than those in the permanent grazing group (Figure S2, Table S3). When all animals included in the study were considered (*n* = 12 per grazing system), mean ADG was 378.0 ± 57.8 g/day in the regenerative group and 242.4 ± 65.8 g/day in the permanent grazing group, representing a significant difference between groups (Welch’s *t*-test, *p* < 0.001). The magnitude of this difference was large, as indicated by a Cohen’s d value of 2.19.

A comparable pattern was observed when the analysis was restricted to the subset of animals for which complete paired fecal samples were available and subsequently included in culture-based and amplicon sequencing analyses (*n* = 8 per grazing system). In this subset, donkeys under regenerative grazing showed a mean ADG of 378.8 ± 69.4 g/day, whereas animals under permanent grazing exhibited a mean ADG of 250.0 ± 56.5 g/day. This difference remained statistically significant (*p* = 0.001), with a similarly large effect size (Cohen’s d = 2.04), indicating that the animals included in the amplicon sequencing analyses showed an ADG pattern comparable to that observed in the full cohort.

Throughout the grazing period, no clinical signs of disease, abnormal behavior, or health-related interventions were recorded, and all donkeys remained in good general health in both grazing groups.

EPG counts were low at both sampling time points in donkeys managed under permanent and regenerative grazing systems, with values ranging from 0 to 150 eggs per gram of feces. Changes in egg counts between sampling times showed no consistent trend and were not indicative of clinically relevant parasitic infection.

### 3.3. Culture-Based Characterization of the Fecal Microbiota

Culture-based fecal microbial counts were evaluated for major microbial groups at the beginning and at the end of the grazing period (Table 3). At the start of grazing, differences in absolute CFU levels were already observable between grazing groups for several microbial categories, reflecting natural inter-individual and group-level variability prior to pasture exposure. During the grazing period, both grazing groups showed comparable overall trends in culture-based microbial counts. Total aerobic and anaerobic bacteria, as well as LAB and *Enterobacteriaceae*, generally showed a decrease in log-transformed CFU values from the start to the end of the grazing period in both grazing systems. In contrast, fungal counts increased over time in both groups.

To account for baseline differences between grazing groups, changes in microbial abundance were further evaluated at the individual level by calculating differences between final and initial CFU values and comparing these between grazing systems using Welch’s *t*-test. While the mean increase in fungal CFU values appeared larger in permanently grazed donkeys compared to the regenerative group, this difference did not reach statistical significance (*p* = 0.140). No statistically significant differences were detected between groups for any of the investigated microbial groups.

### 3.4. Temporal Dynamics of Bacterial and Fungal ASV Diversity

Each grazing-system × sampling-time group comprised samples from eight individual donkeys. After quality filtering, a total of 4,550,843 high-quality bacterial sequences were retained and clustered into 2833 bacterial ASVs. For fungi, 2,353,988 sequences were grouped into 596 fungal ASVs and used for downstream analyses.

Bacterial ASV richness showed contrasting patterns over time between grazing systems (Table S4). Under permanent grazing, the mean number of ASVs in final samples (2435.38 ± 115.54) was only slightly higher than at the start of the grazing period (2401.13 ± 50.25). However, the average number of detected taxa at lower taxonomic levels (class to species) tended to be marginally lower in final_P samples compared to start_P samples. In contrast, bacterial ASV richness increased over time in the regenerative group, with higher mean ASV numbers in final samples (final_R: 2454.00 ± 85.34) compared to the start (start_R: 2325.88 ± 151.07), accompanied by increased taxonomic richness across most taxonomic ranks.

Fungal communities exhibited a clearer temporal change (Table S5). The two start groups displayed highly similar fungal ASV richness under permanent and regenerative grazing (start_P: 282.00 ± 39.02; start_R: 288.38 ± 32.91). In contrast, both final groups were characterized by higher fungal ASV numbers (final_P: 393.50 ± 29.28; final_R: 337.13 ± 29.46), indicating an increase in fungal richness toward the end of the grazing period.

**Figure 1:**
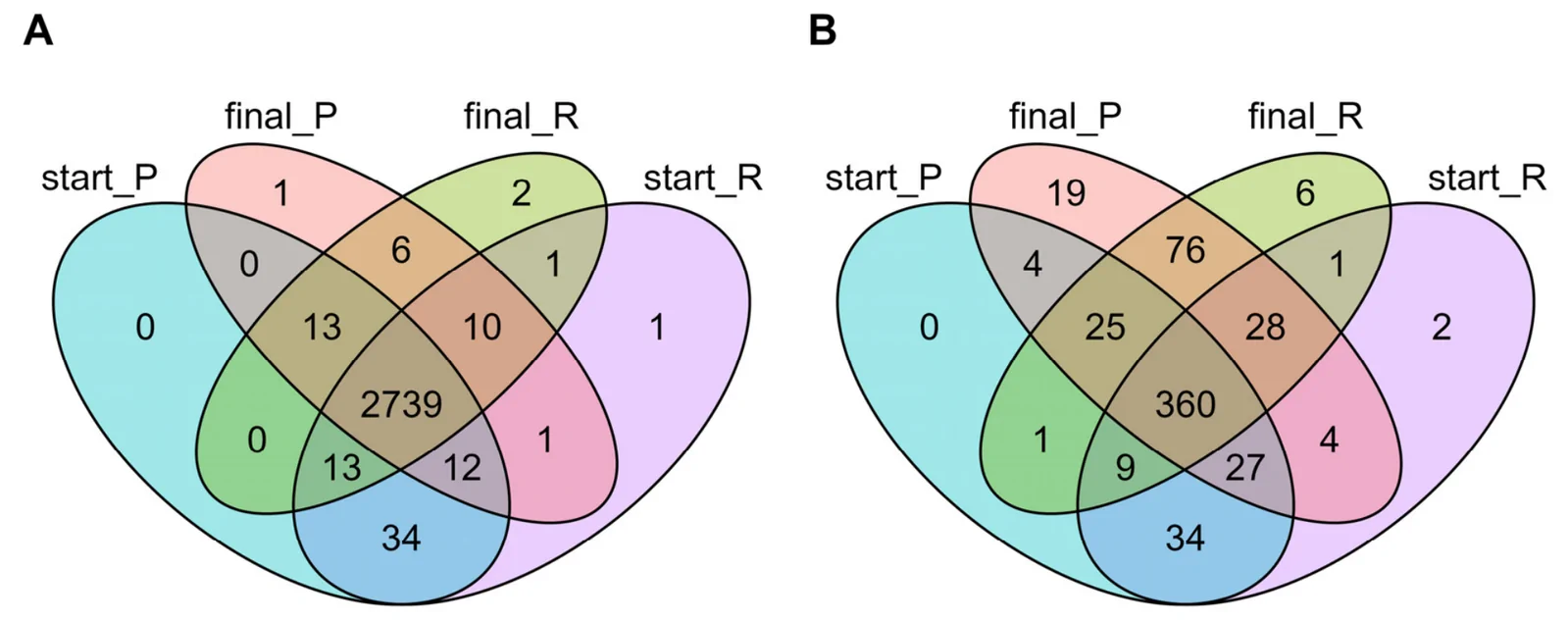
Venn diagrams showing the number of shared and group-specific ASVs identified by NGS for bacterial (**A**) and fungal (**B**) communities across start and final sampling under permanent and regenerative grazing. Group abbreviations: start_P, start samples from the permanent grazing system; start_R, start samples from the regenerative grazing system; final_P, final samples from the permanent grazing system; final_R, final samples from the regenerative grazing system.

Venn diagram analysis indicated that the majority of bacterial ASVs (2739; 96.68%) were shared among all four groups (start and final sampling under permanent and regenerative grazing), consistent with a large core bacterial community (Figure 1). Group-specific bacterial ASVs were relatively few and were more frequently detected in the start samples than in the final samples (35 vs. 9 unique ASVs, respectively), despite the slightly higher overall ASV richness observed at the end of the grazing period. In contrast, fungal communities showed a lower proportion of shared ASVs (360; 60.30%). Group-specific fungal ASVs increased from the start to the final sampling, with 36 unique ASVs detected in the start samples and 101 unique ASVs in the final samples, consistent with the observed increase in fungal ASV richness over time.

### 3.5. Bacterial Community Composition and Diversity

**Figure 2:**
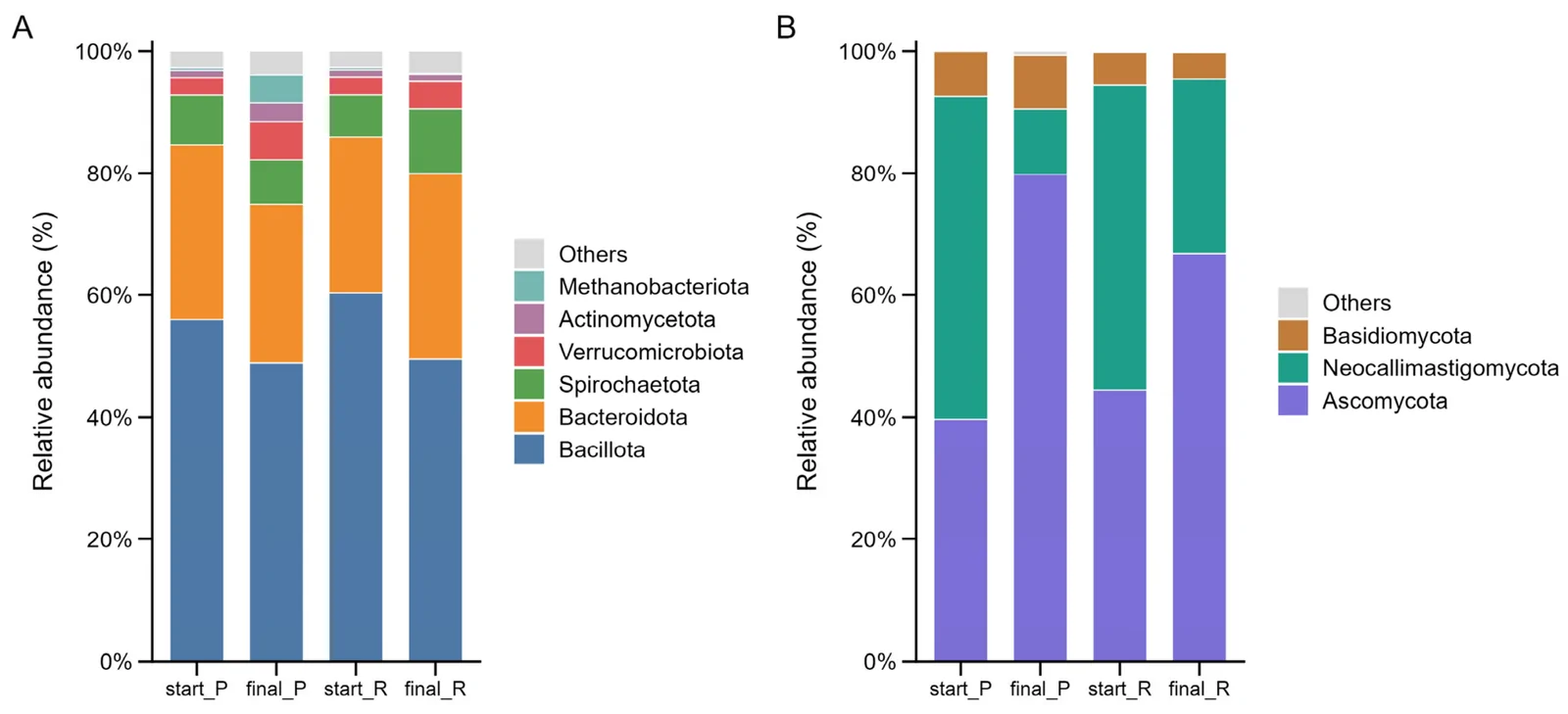
Relative abundance of bacterial (**A**) and fungal (**B**) phyla across grazing systems and sampling times. Phyla with <1% average relative abundance were grouped as “Others”. Group abbreviations: start_P, start samples from the permanent grazing system; start_R, start samples from the regenerative grazing system; final_P, final samples from the permanent grazing system; final_R, final samples from the regenerative grazing system.

**Figure 3:**
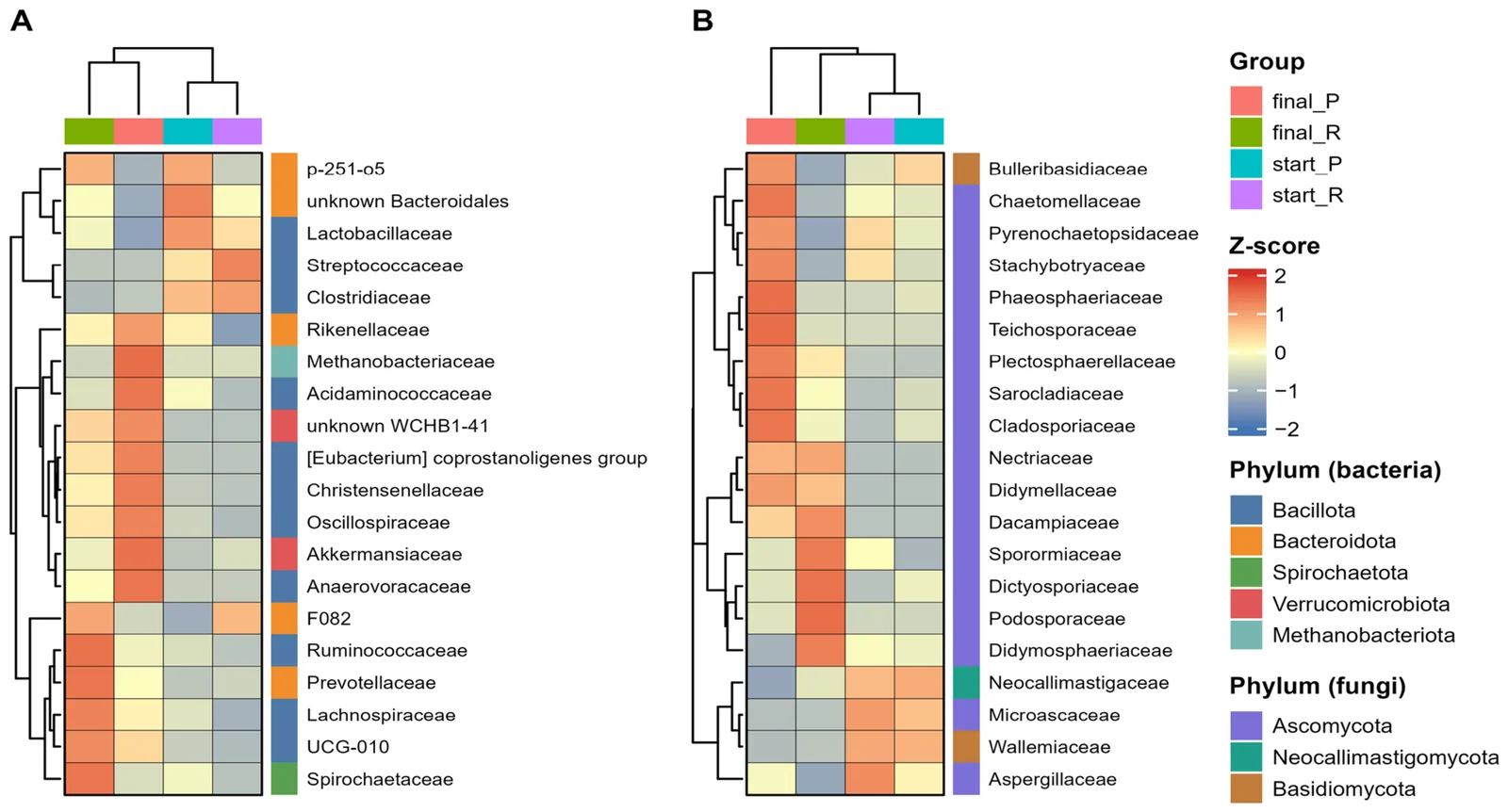
Heatmap of the 20 most abundant bacterial (**A**) and fungal (**B**) families in the fecal samples. Colors represent row-wise Z-scores of relative abundance values. Column annotations indicate grazing group and sampling time: start_P, start samples from the permanent grazing system; start_R, start samples from the regenerative grazing system; final_P, final samples from the permanent grazing system; final_R, final samples from the regenerative grazing system.

A total of 20 phyla, 34 classes, 59 orders, 94 families, 195 genera and 223 species were detected by sequencing (Table S4). Six bacterial phyla exceeded 1% average relative abundance across all samples: Bacillota, Bacteroidota, Spirochaetota, Verrucomicrobiota, Actinomycetota and Methanobacteriota, with average relative abundances of 53.66%, 27.70%, 8.21%, 4.18%, 1.61%, and 1.41%, respectively. All remaining phyla individually accounted for less than 1% of the total bacterial community. Archaea (Methanobacteriota) were detected at low average abundance across samples, with a higher relative mean abundance in the final permanent grazing group (4.62%) than in the other groups (<0.5%). Group-level differences in bacterial community composition at phylum level are shown in Figure 2A, while the relative abundance patterns of the 20 most abundant bacterial families are summarized in the family-level heatmap (Figure 3A).

**Figure 4:**
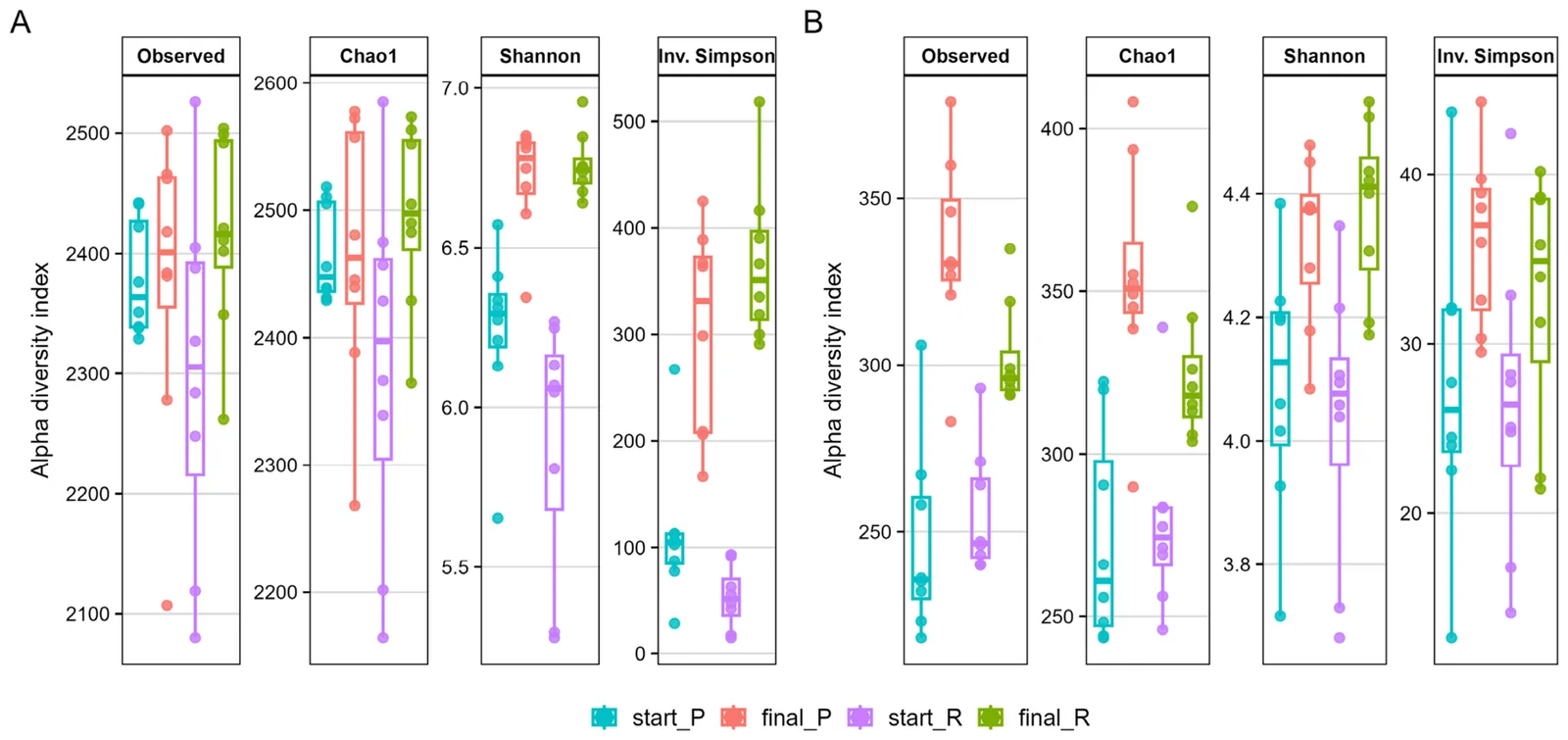
Alpha diversity of bacterial (**A**) and fungal (**B**) communities across grazing systems and sampling times. Alpha diversity was assessed using Observed ASVs, Chao1, Shannon, and inverse Simpson indices. Group abbreviations: start_P, start samples from the permanent grazing system; start_R, start samples from the regenerative grazing system; final_P, final samples from the permanent grazing system; final_R, final samples from the regenerative grazing system.

Alpha diversity of the bacterial communities was assessed using richness-based (Observed ASVs, Chao1) and diversity-based (Shannon, inverse Simpson) indices (Figure 4A). Across the four groups, richness estimates showed relatively modest variation, with overlapping distributions between start and final samples under both grazing systems. In contrast, Shannon and inverse Simpson indices displayed larger differences among groups. In particular, final samples tended to show higher diversity values compared to the corresponding start samples, with different patterns observed between permanent and regenerative grazing systems. To account for the paired sampling design, changes in alpha diversity were further assessed by calculating the difference between final and start samples for each individual and comparing these changes between grazing groups. Changes in richness-based indices (Observed ASVs and Chao1) did not differ significantly between permanent and regenerative grazing (Mann–Whitney U test; raw *p* = 0.105 and 0.065, respectively; BH-adjusted *p* = 0.105 and 0.087, respectively). Changes in diversity indices incorporating evenness were nominally greater under regenerative grazing, as reflected by both the Shannon index (ΔShannon: 0.87 vs. 0.48; raw *p* = 0.038) and the inverse Simpson index (ΔInvSimpson: 314.0 vs. 191.2; raw *p* = 0.038); however, these differences were no longer statistically significant after Benjamini–Hochberg correction (adjusted *p* = 0.076 for both indices).

**Figure 5:**
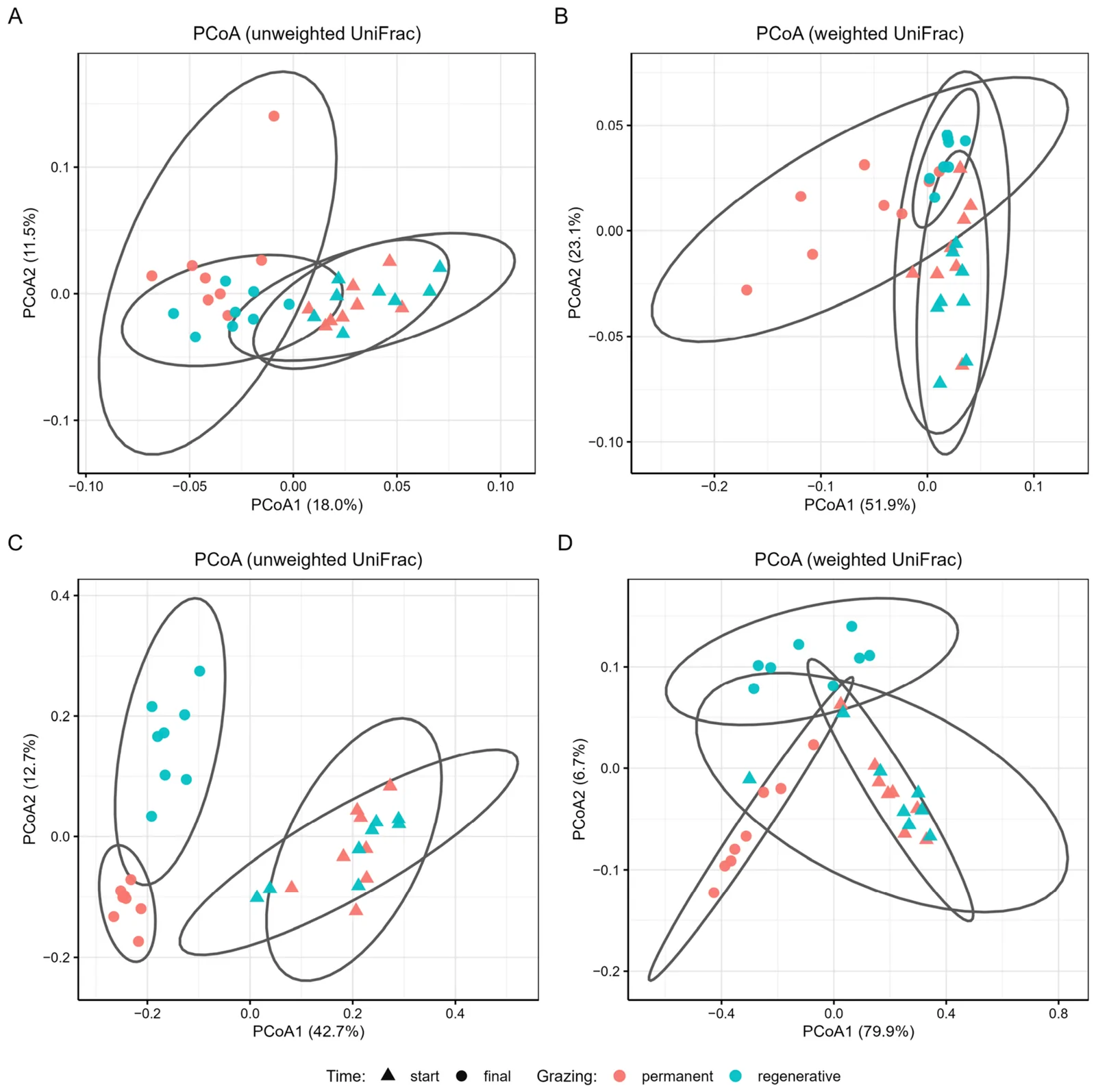
Beta diversity patterns of fecal microbial communities under permanent and regenerative grazing systems. PCoA based on unweighted and weighted UniFrac distance matrices is shown for bacterial (**A**,**B**) and fungal (**C**,**D**) communities. Samples are colored according to grazing system (permanent vs. regenerative) and shaped by sampling time (start vs. final). Ellipses indicate 95% confidence regions for the four grazing system × sampling time groups.

Beta diversity of the bacterial fecal microbiota was assessed using both unweighted and weighted UniFrac distance metrics (Figure 5). PCoA revealed clear structuring of bacterial community composition according to grazing system and sampling time. In particular, weighted UniFrac ordination, which accounts for taxon relative abundances, showed separation between start and final samples, with additional differentiation between permanent and regenerative grazing systems (Figure 5B). In contrast, unweighted UniFrac, reflecting presence-absence patterns, displayed less separation among groups (Figure 5A). PERMANOVA indicated significant differences associated with grazing system (R^2^ = 0.056, F = 2.07, permutation *p* = 0.004) and a significant effect of sampling time (R^2^ = 0.152, F = 5.65, permutation *p* < 0.001) for unweighted UniFrac, whereas their interaction was not significant (R^2^ = 0.042, F = 1.55, permutation *p* = 0.076). For weighted UniFrac, PERMANOVA identified significant associations with grazing system (R^2^ = 0.096, F = 5.03, permutation *p* < 0.001), sampling time (R^2^ = 0.239, F = 12.49, permutation *p* < 0.001), and their interaction (R^2^ = 0.127, F = 6.64, permutation *p* = 0.002). PERMDISP indicated no significant dispersion differences for the unweighted distances, whereas weighted UniFrac showed significant dispersion heterogeneity between grazing systems (F = 4.34, *p* = 0.003) and among the four grazing-by-time groups (F = 7.48, *p* = 0.001), indicating that the corresponding weighted PERMANOVA results should be interpreted with caution.

### 3.6. Fungal Community Composition and Diversity

A total of 6 phyla, 21 classes, 40 orders, 92 families, 172 genera and 282 species were detected by sequencing (Table S5). Three fungal phyla exceeded 1% average relative abundance across all samples: Ascomycota, Neocallimastigomycota and Basidiomycota, with average relative abundances of 57.68%, 35.54% and 6.51%, respectively. All remaining phyla collectively accounted for 0.27% of the total fungal community. Group-level differences in fungal community composition at phylum level are shown in Figure 2B, while the relative abundance patterns of the 20 most abundant fungal families are summarized in the family-level heatmap (Figure 3B).

Fungal alpha diversity exhibited clear temporal changes across both grazing systems (Figure 4B). Across the four groups, richness estimates showed increases from start to final sampling under both grazing systems, although variability was observed among individuals. Diversity indices incorporating evenness displayed more modest shifts, with overlapping distributions between start and final samples in both permanent and regenerative grazing groups. Paired changes in fungal alpha diversity between start and final sampling revealed a tendency toward larger increases under permanent grazing, particularly for observed ASVs, although these differences were not statistically significant (ΔObserved: 87.8 vs. 47.0; Mann–Whitney U test, *p* = 0.065). No significant differences were detected between the two grazing groups for Chao1, Shannon, or inverse Simpson indices (*p* > 0.2 for all comparisons). The absence of significant differences was unchanged after Benjamini–Hochberg correction.

Fungal beta diversity showed pronounced temporal structuring, with additional differences associated with grazing systems that depended on the distance metric used (Figure 5C,D). PCoA indicated clustering of samples according to grazing groups and sampling time, with separation along the first principal coordinate in the weighted UniFrac ordination, reflecting substantial differences in the dominant fungal taxa among groups. For unweighted UniFrac, PERMANOVA detected significant variation associated with grazing system (R^2^ = 0.072, F = 4.33, permutation *p* < 0.001), sampling time (R^2^ = 0.391, F = 23.60, permutation *p* < 0.001), and their interaction (R^2^ = 0.072, F = 4.36, permutation *p* = 0.044). However, dispersion differed significantly among the four grazing-by-time groups (PERMDISP: F = 10.45, *p* < 0.001), requiring cautious interpretation of these group-level differences. In the weighted UniFrac analysis, sampling time was the dominant effect (R^2^ = 0.463, F = 31.79, permutation *p* < 0.001), whereas neither grazing system (R^2^ = 0.055, F = 3.75, permutation *p* = 0.063) nor the grazing-by-time interaction (R^2^ = 0.074, F = 5.05, permutation *p* = 0.059) reached statistical significance. No significant dispersion heterogeneity was detected for weighted UniFrac.

### 3.7. Longitudinal Differential Abundance Analysis of Bacterial Communities

**Figure 6:**
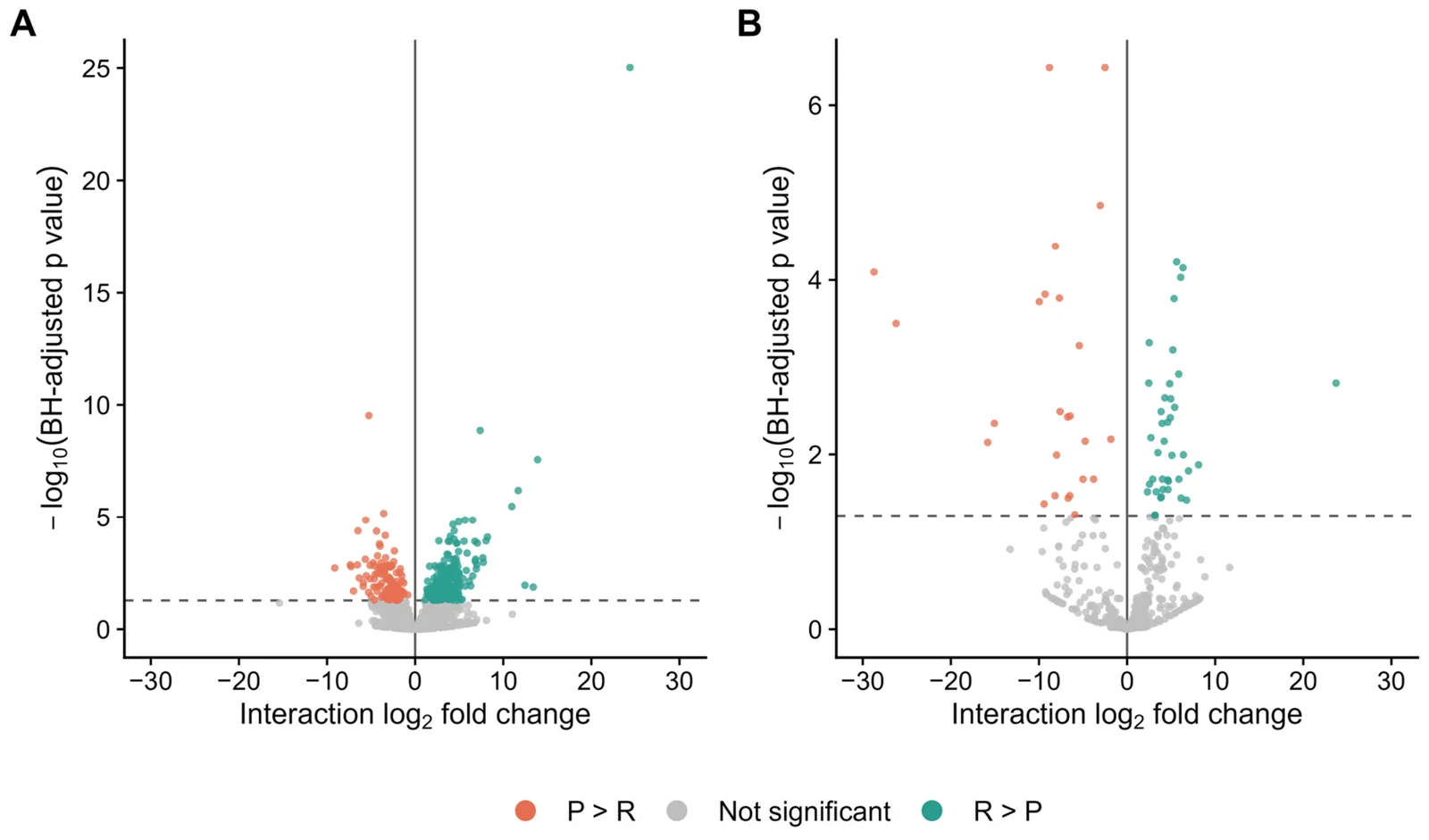
Longitudinal differential-abundance analysis of bacterial (**A**) and fungal (**B**) ASVs based on the grazing system × sampling time interaction. Volcano plots show interaction log_2_ fold changes from the DESeq2 model, representing the difference in temporal change between regenerative and permanent grazing systems, plotted against -log_10_ Benjamini–Hochberg-adjusted *p*-values. Each point represents a single ASV. Red points indicate ASVs with a more positive temporal change under permanent grazing than under regenerative grazing, whereas green points indicate ASVs with a more positive temporal change under regenerative grazing than under permanent grazing. Grey points represent non-significant ASVs. The dashed horizontal line indicates the significance threshold (adjusted *p* = 0.05).

Longitudinal differential abundance analysis identified 336 of the 2833 bacterial ASVs (11.86%) with a significant grazing system × sampling time interaction after Benjamini–Hochberg correction (adjusted *p* < 0.05; Tables S6 and S7). Of these, 195 ASVs had positive interaction coefficients, indicating a more positive start-to-final change under regenerative grazing relative to permanent grazing, whereas 141 had negative interaction coefficients, indicating the opposite pattern. The distribution of interaction log_2_ fold changes and adjusted *p*-values is shown in Figure 6A. Among the significant interaction ASVs, all 17 ASVs affiliated with Spirochaetota showed positive interaction coefficients, whereas three *Methanobrevibacter* ASVs showed negative interaction coefficients.

**Figure 7:**
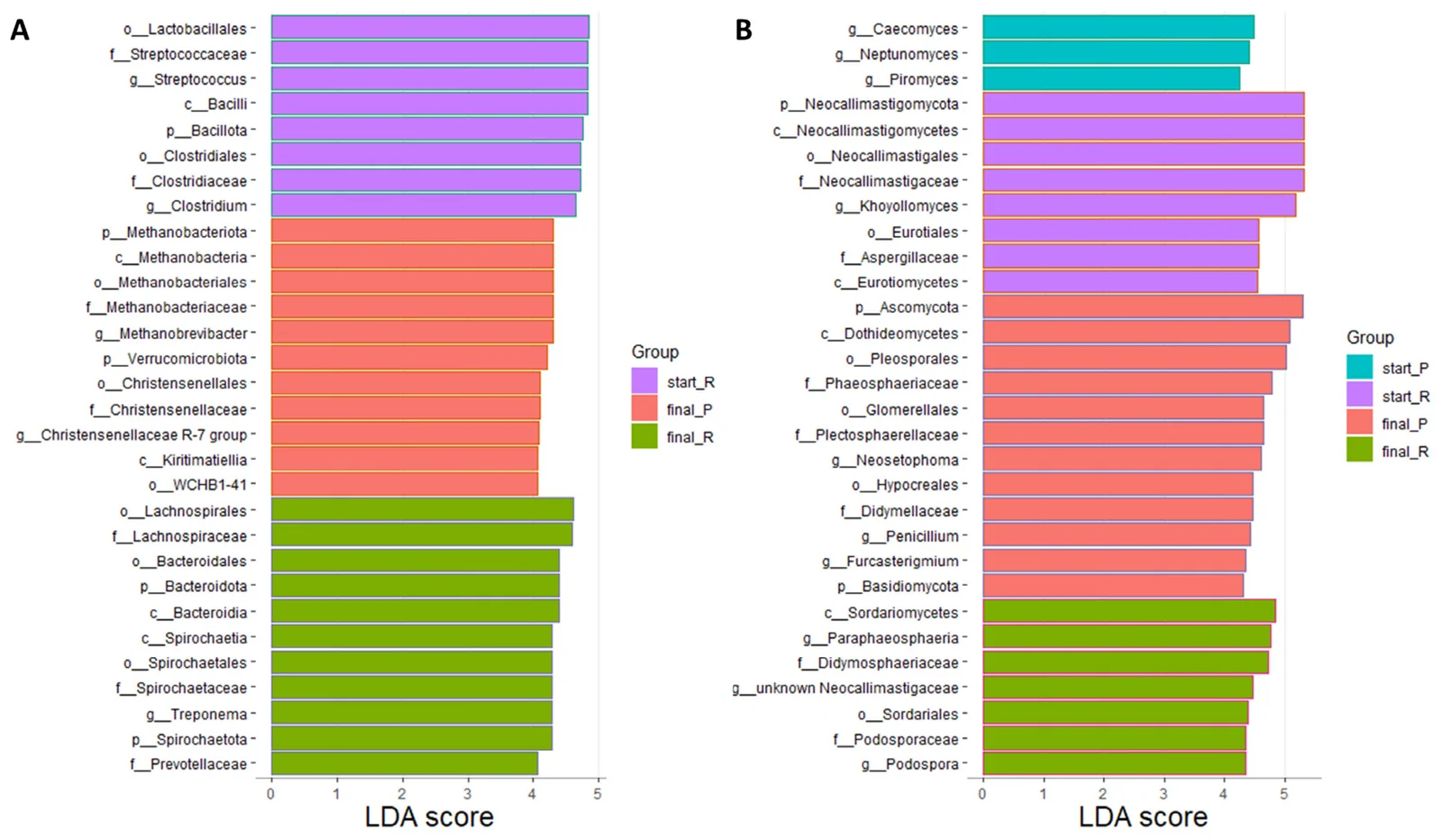
LEfSe analysis of microbial community biomarkers across grazing systems and sampling times. The top 30 bacterial (**A**) and fungal (**B**) taxa discriminating between grazing systems and sampling time points were identified based on LDA scores. Taxa are shown across multiple taxonomic ranks and colored according to the group in which they were enriched. Taxonomic prefixes indicate phylum (p__), class (c__), order (o__), family (f__), and genus (g__). Group abbreviations: start_P, start samples from the permanent grazing system; start_R, start samples from the regenerative grazing system; final_P, final samples from the permanent grazing system; final_R, final samples from the regenerative grazing system.

Complementary taxonomic patterns across grazing systems and sampling times were further explored using LEfSe analysis. LEfSe identified distinct bacterial taxa differing among grazing-system and sampling-time groups, with differences observed across multiple taxonomic ranks (Figure 7A). Final_P was characterized by enrichment of methanogenic archaea and associated taxa, including members of Methanobacteriota (e.g., Methanobacteria, Methanobacteriales, *Methanobacteriaceae* and *Methanobrevibacter*), as well as taxa affiliated with Verrucomicrobiota, including the poorly characterized WCHB1-41 taxon, and members of Christensenellales. In contrast, final_R was associated with taxa affiliated with Bacteroidota (Bacteroidia, Bacteroidales) and Spirochaetota (Spirochaetales and *Treponema*), while additional biomarkers were affiliated with Lachnospirales/*Lachnospiraceae* and *Prevotellaceae*. At the start of the grazing period, LEfSe biomarkers were dominated by Bacillota-related taxa, particularly members of Lactobacillales, *Streptococcaceae* (*Streptococcus*), and Clostridiales (*Clostridiaceae*, *Clostridium*).

### 3.8. Longitudinal Differential Abundance Analysis of Fungal Communities

Longitudinal differential abundance analysis identified 66 of the 596 fungal ASVs (11.07%) with a significant grazing system × sampling time interaction (adjusted *p* < 0.05; Table S6). Adjusted *p*-values were available for 480 fungal ASVs after DESeq2 independent filtering. Of the significant interaction ASVs, 41 had positive interaction coefficients and 25 had negative interaction coefficients. The distribution of interaction log_2_ fold changes and adjusted *p*-values is shown in Figure 6B. Notably, 31 significant interaction ASVs were affiliated with Neocallimastigomycota, and all showed positive interaction coefficients, indicating a more positive temporal change under regenerative grazing relative to permanent grazing.

LEfSe revealed distinct fungal taxa associated with grazing system and sampling time, with biomarker patterns observed across several taxonomic ranks (Figure 7B). At the start of the grazing period, LEfSe identified distinct fungal biomarkers between groups: start_R was characterized by enrichment of Neocallimastigomycota (Neocallimastigomycetes, Neocallimastigales and *Neocallimastigaceae*), including *Khoyollomyces*, whereas start_P showed genus-level biomarkers such as *Caecomyces* and *Piromyces*. In contrast, fungal biomarkers characterizing final_P samples were predominantly affiliated with Ascomycota, including taxa within Dothideomycetes and Pleosporales (e.g., *Phaeosphaeriaceae*, *Plectosphaerellaceae* and *Neosetophoma*), as well as representatives of Hypocreales and Eurotiales. Final_R samples were characterized by taxa belonging to Sordariomycetes, including *Paraphaeosphaeria*, *Didymosphaeriaceae*, *Podosporaceae* and *Podospora*.

Because the four-group LEfSe analysis was largely driven by temporal variation between start and final samples, an additional targeted LEfSe comparison was performed between final_P and final_R samples to highlight end-point-specific fungal biomarkers (Figure S3). This focused endpoint analysis provided complementary taxonomic information, showing a higher representation of anaerobic gut-associated fungi (Neocallimastigomycota) in final_R samples, whereas final_P samples were characterized by a greater relative abundance of taxa affiliated with Ascomycota.

## 4. Discussion

### 4.1. Weather and Forage Conditions During the Grazing Period

Weather and forage conditions provided an important background for interpreting the grazing-related responses observed in this study. Before the start of grazing, all donkeys had been maintained under the same stable conditions and received the same grass hay diet for five months, providing a common nutritional background. At the first vegetation sampling, shortly before pasture access, the measured forage dry matter per unit area and nutrient composition were broadly similar between the two pasture areas, indicating comparable initial forage conditions.

During the grazing period, the study area experienced a marked rainfall deficit, with June being the most affected month. Only 3 mm of precipitation was recorded in June, compared with the 1991–2021 reference value of 85 mm, and only 10 mm of July precipitation occurred while animals were still on pasture. These dry conditions were reflected in the forage composition. By mid-June, moisture content had declined markedly in both pastures, while crude protein decreased and fiber fractions increased. Crude protein declined from approximately 125 g/kg DM at the start of grazing to 70 g/kg DM in the permanent pasture and 86 g/kg DM in the regenerative pasture, whereas NDF increased to about 610 g/kg DM in both pastures. These changes indicate progressive drying and maturation of the available forage during the grazing period.

Despite the shared weather conditions, the two grazing systems differed in pasture use. Under permanent grazing, animals had continuous access to the whole pasture, while in the regenerative system, previously grazed paddocks were allowed to rest before being grazed again. The June samples from the regenerative pasture represented paddocks with approximately 42 days of regrowth, and these samples retained significantly higher moisture content (58.94%) than the permanent pasture samples (48.89%). These forage conditions provide an important context for interpreting the subsequent differences in animal performance and fecal microbial community structure.

### 4.2. Animal Performance Under Different Grazing Systems

Donkeys managed under regenerative grazing exhibited higher ADG over the 82-day weighing interval compared to animals under permanent grazing (378.0 ± 57.8 vs. 242.4 ± 65.8 g/day). Although animal growth performance was not the primary focus of the study and the number of individuals was limited, these results offer an important physiological context for interpreting grazing-related microbial patterns.

Evidence directly linking grazing management to animal growth performance is surprisingly scarce, particularly outside intensively managed ruminant systems. Most studies addressing regenerative, adaptive multi-paddock, or rotational grazing have focused on vegetation structure, forage availability, soil carbon dynamics, and ecosystem services, rather than direct measurements of animal weight gain or feed efficiency [13,14,15,17]. Where animal-level responses are discussed, they are often inferred indirectly through increased standing biomass or carrying capacity rather than quantified growth outcomes. Comparable data for equids are even more limited, as donkey research has largely concentrated on nutrition, health, and work performance under controlled feeding conditions rather than extensive pasture-based systems [34,62].

In this study, the higher ADG observed in the regenerative group is consistent with the forage conditions recorded during the dry grazing period. The rotational use of paddocks allowed previously grazed areas to regrow before being used again, and the June forage data indicated higher moisture content in these regrowth paddocks than in the continuously grazed pasture. These observations indicate differences in forage conditions between the two pastures during a period when rainfall was strongly limited and vegetation drying was progressing in both pastures. As hindgut fermenters, donkeys may respond to such differences in forage structure and availability differently from ruminants, and the present results provide field-based evidence that growth performance differed between the two observed pasture-management contexts under extensive grazing conditions.

### 4.3. Fecal Microbial Community Responses to Grazing Management

Culture-based fecal microbial counts did not show significant differences between grazing systems, whereas amplicon sequencing revealed clear differences in bacterial and fungal fecal community structure. This contrast is not unexpected, because plate counts describe broad cultivable groups, while sequencing captures compositional shifts across a much larger fraction of the microbial community. In the present study, the two grazing groups did not differ significantly in total cultivable microbial loads, whereas differences were detected in the internal structure of the fecal microbiota.

The bacterial data suggest that differences between the two pasture-management contexts were expressed mainly as relative-abundance shifts within a largely shared bacterial community. Across all groups, 96.7% of bacterial ASVs were shared, indicating a large bacterial core that persisted across grazing systems and sampling times. This agrees with previous equine and donkey studies showing that hindgut bacterial communities often retain a conserved taxonomic backbone, while diet- or management-related effects are expressed mainly through shifts in the relative abundance of dominant taxa [27,30,35,63]. In the present study, this pattern was also reflected in alpha diversity: richness-based indices changed only modestly, whereas evenness-sensitive indices (Shannon and inverse Simpson) showed larger numerical increases under regenerative grazing. Although these differences did not remain statistically significant after correction for multiple testing, they should therefore be interpreted as trends toward greater bacterial evenness rather than as evidence of a significant between-group difference in alpha diversity.

The fungal community showed a different pattern. Only 60.3% of fungal ASVs were shared across all groups, and the number of group-specific fungal ASVs increased from 36 at the start of grazing to 101 at the final sampling. Fungal richness also increased toward the end of the grazing period in both grazing systems. These results indicate stronger temporal turnover in fungal communities than in bacterial communities. This is consistent with the ecology of gut-associated fungi, especially anaerobic fungi, which respond to changes in plant substrate availability and forage structure [40,41,42]. Donkey-focused studies also suggest that fungal communities are more variable than bacterial communities and may be sensitive to feeding strategy and gut environment [27,38].

Beta diversity analyses indicated that both grazing system and sampling time were associated with bacterial community differentiation, although the strength of these associations depended on the distance metric. Sampling time showed a consistent effect in both unweighted and weighted UniFrac analyses, while the grazing-by-time interaction was significant only for weighted UniFrac. The stronger effects observed for weighted UniFrac suggest that changes in the relative abundance of phylogenetically related taxa contributed substantially to bacterial community differentiation. However, significant dispersion heterogeneity was also detected for weighted UniFrac, and the corresponding PERMANOVA results should therefore be interpreted with caution. This is in line with previous work showing that diet and forage type often influence herbivore gut microbiota through shifts in dominant groups [20,21,64,65]. Fungal beta diversity showed an even stronger temporal component. Sampling time accounted for the largest proportion of variation in both UniFrac analyses, whereas the association with grazing system and grazing-by-time interaction were less consistent between distance metrics. Unweighted UniFrac also showed significant dispersion heterogeneity among grazing-by-time groups, limiting a simple centroid-based interpretation of these differences. Overall, bacterial and fungal communities displayed different patterns of change: bacterial differentiation was more closely associated with shifts within a largely shared community, whereas fungal communities showed stronger temporal restructuring and compositional turnover.

These findings should also be interpreted in the forage context described above. Differences in forage availability and structure provide a plausible ecological context for the observed microbial patterns. Previous equine and donkey studies have shown that forage type, fiber content, feeding strategy, and physiological state can shape fecal microbial composition [31,64,65,66]. The present results extend this concept to a field-based grazing system, where microbial differences reflected the combined variation in pasture use, forage dynamics, and seasonal drying rather than a single controlled dietary factor.

### 4.4. Taxonomic Signatures of Permanent and Regenerative Grazing

At higher taxonomic levels, the fecal bacterial community was consistent with previous descriptions of equine and donkey hindgut microbiota. Bacillota and Bacteroidota dominated across samples, together accounting for most bacterial sequences. These phyla are commonly reported in equids maintained on forage-based diets and include many taxa involved in carbohydrate fermentation and plant fiber degradation [26,35,36,64]. Spirochaetota and Verrucomicrobiota were also consistent secondary components of the bacterial community. Members of Spirochaetota, including *Treponema*-related taxa, are frequently detected in herbivore hindgut systems and have been linked to the degradation of complex plant-derived polysaccharides [24,37,38]. LEfSe also identified distinct bacterial and fungal biomarkers between start_P and start_R before pasture exposure. These baseline differences indicate appreciable between-group variability and therefore caution against attributing endpoint LEfSe differences solely to grazing management. The longitudinal DESeq2 analysis addresses this issue more directly by testing whether start-to-final changes differed between the two grazing groups rather than relying on endpoint comparisons alone.

Among the archaeal sequences recovered using the 16S rRNA primer set, Methanobacteriota was the predominant phylum. Although its average relative abundance was low, Methanobacteriota increased in the final permanent grazing group, where *Methanobrevibacter* and related methanogenic taxa were identified among the final_P-associated biomarkers. The longitudinal DESeq2 analysis further identified three *Methanobrevibacter* ASVs with significant negative grazing system × sampling time interactions, indicating a more positive temporal change under permanent grazing relative to regenerative grazing. Methanogenic archaea act as hydrogen consumers in anaerobic fermentation systems and are part of the broader microbial network involved in plant fiber degradation [21,67,68]. Together, these findings suggest a distinct temporal pattern between the grazing groups among the detected methanogenic archaeal taxa; however, this should not be interpreted as a comprehensive response of the archaeal community because the primers were not specifically optimized for archaeal profiling.

The longitudinal differential-abundance analysis also identified contrasting temporal responses between grazing groups among bacterial taxa involved in hindgut fermentation. Notably, all 17 significant interaction ASVs affiliated with Spirochaetota showed positive interaction coefficients, indicating a more positive temporal change under regenerative grazing relative to permanent grazing. Endpoint LEfSe analysis additionally associated final_R samples with Bacteroidota and Spirochaetota, including *Treponema* and Lachnospirales. These groups include taxa capable of degrading plant polysaccharides and fermenting complex carbohydrates, and their temporal and endpoint patterns are consistent with a fecal community shaped by forage-based hindgut fermentation [19,24,64,69,70]. Given the higher moisture content of regrowth forage in the regenerative paddocks, these bacterial patterns are compatible with differences in forage structure and availability between grazing systems. This does not imply that the detected taxa directly caused the higher ADG, but the animal-performance and microbial patterns occurred alongside the observed differences in forage context.

Fungal communities were dominated by Ascomycota and Neocallimastigomycota, with Basidiomycota present at lower relative abundance. The high representation of Neocallimastigomycota is notable because anaerobic gut fungi play a central role in the breakdown of lignocellulose-containing plant material through both enzymatic activity and physical penetration of plant tissues [40,41,42]. Although anaerobic fungi have been studied most extensively in ruminants, they are also present in non-ruminant herbivores, including equids, and recent work indicates that they form an important part of the donkey hindgut ecosystem [27,38].

The fungal differential abundance results showed a contrast between anaerobic gut-associated fungi and other fungal taxa. Longitudinal DESeq2 identified 31 significant interaction ASVs affiliated with Neocallimastigomycota, all with positive interaction coefficients, indicating a more positive temporal change under regenerative grazing relative to permanent grazing. Importantly, this pattern reflected differences in temporal trajectories rather than simply higher abundance at the final sampling. The targeted endpoint LEfSe analysis provided complementary evidence of a higher representation of Neocallimastigomycota in final_R samples. This pattern is consistent with the role of anaerobic fungi in processing fibrous plant material in the hindgut. Similar links between forage conditions, anaerobic fungi, and equine gut microbial communities have been reported in recent studies on seasonal forage and dietary fiber effects [70].

In contrast, final_P samples were more strongly associated with Ascomycota-affiliated taxa, including members of Dothideomycetes, Pleosporales, Hypocreales, and Eurotiales. These associations were identified primarily in the endpoint LEfSe analysis and should therefore be interpreted as taxonomic patterns at the final sampling rather than as evidence of differential temporal change. Many Ascomycota detected in herbivore feces are likely to include plant-associated, saprotrophic, or environmentally derived fungi passing through the gastrointestinal tract with ingested plant material. Their presence does not necessarily indicate stable colonization of the hindgut. This distinction is important because fecal ITS profiles may reflect both resident gut-associated fungi and fungi derived from the consumed vegetation or surrounding environment. Segment-specific studies in equids also support the idea that fungal communities differ along the gastrointestinal tract, with anaerobic fungal groups being more closely linked to hindgut fermentation than many non-anaerobic fungal taxa [71]. Therefore, the higher representation of Ascomycota in final_P samples may reflect differences in ingested plant material, pasture surface exposure, or transient environmental fungi, whereas the more positive temporal response of Neocallimastigomycota in the regenerative group is more consistent with differences in fiber-associated hindgut fermentation.

Overall, the taxonomic results show that the two pasture-management contexts were associated with distinct temporal patterns in bacterial and fungal ASVs, together with differences among the archaeal taxa detected by the 16S assay. More positive temporal responses were observed in the regenerative group among several bacterial taxa linked to plant polysaccharide fermentation and among Neocallimastigomycota ASVs, whereas detected *Methanobrevibacter* ASVs showed more positive temporal changes under permanent grazing. Endpoint LEfSe patterns additionally distinguished the two grazing systems at the final sampling. These patterns support a multi-domain interpretation of the fecal microbiota, with differences observed in bacterial and fungal community structure as well as among the archaeal taxa recovered by the 16S assay, alongside the differing forage conditions of the two pastures.

### 4.5. Study Implications and Limitations

The present study demonstrates the value of sequencing-based approaches for characterizing fecal microbial variation in donkeys under field grazing conditions. Although culture-based microbial counts showed no significant differences between groups, amplicon sequencing revealed temporal and between-group differences in bacterial and fungal community structure. The inclusion of fungal communities was particularly informative, as fungal ASV turnover and Neocallimastigomycota-associated patterns provided complementary insight into fiber-associated hindgut processes. More broadly, the study extends grazing-related microbiome research to donkeys, a hindgut-fermenting herbivore that remains underrepresented compared with ruminant livestock.

Several limitations should be considered. Most importantly, each grazing management was represented by a single pasture; therefore, pasture identity and management were confounded and no independent pasture-level replication was available. Individual donkeys represented animal-level observational units within these two pasture-management contexts rather than independent replicates of the grazing treatment. Consequently, the observed differences in animal performance, forage characteristics, and microbiota should be interpreted as contrasts between the two pasture-management contexts and not as causal or broadly generalizable effects of grazing management.

The microbiome analyses were based on eight animals per group with complete paired fecal samples, which limited the assessment of inter-individual variability. In addition, the grazing period coincided with an unusually dry period that strongly restricted pasture regrowth and led to earlier termination of grazing than originally planned. These drought conditions may have influenced forage availability and quality and may have altered the magnitude of the observed differences between the two pasture-management contexts. In particular, the higher ADG observed in the regenerative group should therefore not be attributed to grazing management alone. Moreover, ADG was calculated over the 82-day interval between body weight measurements, whereas the animals spent 70 days on pasture, and should not be interpreted as growth performance exclusively during the grazing period.

The vegetation measurements provided contextual information on forage conditions but did not directly quantify the diet consumed by individual animals, and the second vegetation sampling preceded the final fecal sampling by approximately one month. Finally, the 16S rRNA primer set was primarily intended for bacterial profiling and was not optimized for comprehensive archaeal coverage; therefore, the detected archaeal patterns should be interpreted as observations for the taxa recovered by this assay rather than as a quantitative representation of the complete archaeal community.

## 5. Conclusions

This study shows that the fecal microbial communities of donkeys changed over the grazing period and exhibited differences between the two pasture-management contexts. Although culture-based microbial counts did not differ between grazing groups, amplicon sequencing revealed a consistent temporal component in bacterial and fungal community structure, while the statistical support for grazing-group differences and the grazing system × sampling time interaction depended on the distance metric. Longitudinal differential abundance analysis further identified bacterial and fungal ASVs whose start-to-final changes differed significantly between grazing groups. Differences were also observed among the archaeal taxa detected by the 16S assay, but these findings should be interpreted cautiously.

Bacterial communities were characterized by a largely shared core across grazing groups, while longitudinal analysis identified contrasting temporal responses between grazing groups among individual bacterial ASVs, including Spirochaetota and detected *Methanobrevibacter* taxa. Fungal communities showed greater compositional plasticity, with particularly pronounced between-group differences in temporal response among Neocallimastigomycota ASVs. Together, these patterns highlight the importance of considering multiple microbial domains when evaluating fecal microbial patterns under contrasting grazing-management contexts in hindgut-fermenting herbivores.

By integrating bacterial and fungal community analyses in an underrepresented host species, this study provides new insight into fecal microbial ecology under contrasting grazing-management contexts in a non-ruminant herbivore. The findings underscore the value of sequencing-based approaches for detecting microbial differences associated with management context that are not captured by conventional microbiological methods and show that the regenerative group displayed distinct temporal responses among microbial taxa linked to fiber-associated fermentation processes. Overall, within the scope of this field comparison, this work contributes to a more comprehensive understanding of associations among grazing-management context, fecal microbiota, and herbivore-associated ecosystem processes in extensive grassland systems.

## Figures and Tables

**Table 1:** Weather conditions during the study period compared with long-term reference values.

Month (2025)	Temperature (°C)	Precipitation (mm)
April	11.7 (12.2)	28 (58)
May	13.3 (16.8)	30 (79)
June	20.5 (20.7)	3 (85)
July	21.4 (22.6)	38 * (72)

Monthly mean temperature (°C) and precipitation (mm) data from April to July 2025 in the study area. Long-term monthly reference values for the 1991–2021 period, provided by HungaroMet, are shown in parentheses. * Only 10 mm of July precipitation occurred while animals were still on pasture.

**Table 2:** Comparison of the nutrient components of forage from permanent and regenerative pastures.

Forage Parameter	4 May Permanent (*n* = 6)	4 May Regenerative (*n* = 6)	18 June Permanent (*n* = 6)	18 June Regenerative (*n* = 3)
DM (g/0.25 m^2^)	134.92 ± 9.11 a	115.2 ± 14.48 a	195.73 ± 14.7 a	152.53 ± 17.5 a
Moisture content (%)	78.26 ± 0.72 a	79.43 ± 1.32 a	48.89 ± 1.9 a	58.94 ± 2.31 b
Crude protein (g/kg DM)	126.2 ± 4.6 a	123.8 ± 3.7 a	69.8 ± 2.6 a	85.7 ± 11.5 a
Crude fat (g/kg DM)	32.2 ± 1.7 a	32.2 ± 1.1 a	21.5 ± 0.7 a	26.2 ± 2.2 b
Crude fiber (g/kg DM)	261.8 ± 4.4 a	251.1 ± 14.6 a	320 ± 6.9 a	318.4 ± 25.5 a
Crude ash (g/kg DM)	103.8 ± 5.1 a	105.9 ± 5.5 a	88.7 ± 3.1 a	90.6 ± 6.9 a
NDF (g/kg DM)	524.4 ± 9.7 a	499.5 ± 29.3 a	611.4 ± 9.2 a	611.8 ± 42.9 a
ADF (g/kg DM)	308.8 ± 6.7 a	294.3 ± 19.2 a	376.2 ± 7.3 a	368.9 ± 36.3 a
ADL (g/kg DM)	45.7 ± 3.9 a	37.8 ± 4.5 a	45.5 ± 2.5 a	48.6 ± 6.9 a
Hemicellulose (g/kg DM)	215 ± 4.3 a	205.2 ± 14.4 a	234.2 ± 5.2 a	245.4 ± 9.3 a
Cellulose (g/kg DM)	263.1 ± 5.6 a	258.1 ± 17.4 a	330.6 ± 7.7 a	320.3 ± 29.4 a

Values are shown as mean ± standard error. Moisture content is expressed as %, whereas nutrient concentrations are expressed as g/kg DM. Different letters indicate statistically significant differences between pastures within the same sampling month. DM, dry matter; NDF, neutral detergent fiber; ADF, acid detergent fiber; ADL, acid detergent lignin.

**Table 3:** Culture-based microbial counts in donkey fecal samples.

Group	Aerobic Bacteria	Anaerobic Bacteria	LAB	*Enterobacteriaceae*	Aerobic Fungi
start_P	7.55 ± 0.30	7.90 ± 0.20	7.59 ± 0.26	5.52 ± 0.66	2.47 ± 0.53
start_R	7.76 ± 0.42	8.20 ± 0.37	7.95 ± 0.22	6.27 ± 0.61	3.08 ± 0.60
final_P	7.00 ± 0.47	6.96 ± 0.28	6.55 ± 0.46	5.30 ± 1.07	3.73 ± 0.28
final_R	7.17 ± 0.61	7.43 ± 0.48	6.64 ± 0.72	6.16 ± 0.73	3.87 ± 0.15

Mean (± SD) log_10_-transformed culture-based microbial counts (CFU/g wet weight) determined in donkey fecal samples collected before the start of grazing (start) and at the termination of the grazing period (final) under permanent (P) and regenerative (R) grazing systems (*n* = 8 per group).

## Data Availability

The raw sequencing data have been deposited in the NCBI Sequence Read Archive (SRA) under BioProject accession number PRJNA1422485. The data supporting the findings of this study are included within the article and its Supplementary Materials.

## Supplementary Materials

The following supporting information can be downloaded at https://www.mdpi.com/article/10.3390/ani16203288/s1, Figure S1: Daily weather conditions during the grazing period; Figure S2: Average daily gain of donkeys under permanent and regenerative grazing systems; Figure S3: LEfSe analysis comparing fungal communities between final_P and final_R samples, highlighting end-point-specific fungal biomarkers; Table S1: Nutritional composition of grass hay provided to the animals prior to the grazing period; Table S2: Stocking density and total live weight of animals in the two grazing systems; Table S3: Individual body weight gain and average daily gain of donkeys under regenerative and permanent grazing systems; Table S4: 16S rRNA gene amplicon sequencing summary and taxonomic composition; Table S5: ITS gene amplicon sequencing summary and taxonomic composition; Table S6: Summary of longitudinal DESeq2 differential-abundance analysis based on the grazing system × sampling time interaction; Table S7: Complete ASV-level results for significant grazing system × sampling time interactions from the longitudinal DESeq2 analysis.

## Abbreviations

The following abbreviations are used in this manuscript:

16S rRNA	16S ribosomal RNA
ADF	Acid Detergent Fiber
ADG	Average Daily Gain
ADL	Acid Detergent Lignin
ANOVA	Analysis of variance
ASV(s)	Amplicon Sequence Variant(s)
BLAST	Basic Local Alignment Search Tool
CFU	Colony Forming Unit
DM	Dry Matter
EPG	Eggs Per Gram
final_P	Final sampling, permanent grazing system
final_R	Final sampling, regenerative grazing system
FROGS	Find, Rapidly, OTUs with Galaxy Solution
ITS	Internal Transcribed Spacer
LAB	Lactic Acid Bacteria
LDA	Linear Discriminant Analysis
LEfSe	Linear Discriminant Analysis Effect Size
NCBI	National Center for Biotechnology Information
NDF	Neutral Detergent Fiber
NGS	Next-Generation Sequencing
PCoA	Principal Coordinates Analysis
PCR	Polymerase Chain Reaction
PERMANOVA	Permutational Multivariate Analysis of Variance
R^2^	Coefficient of Determination
SD	Standard Deviation
SE	Standard Error
SRA	Sequence Read Archive
start_P	Start sampling, permanent grazing system
start_R	Start sampling, regenerative grazing system
UniFrac	Unique Fraction
