# Supplementary material for: Permanent and Regenerative Grazing Systems Are Associated with Distinct Fecal Bacterial and Fungal Microbiota Patterns in Donkeys Under Extensive Pasture Conditions
Source: Animals (Basel). 2026 Oct 19;16(20):3288. doi: 10.3390/ani16203288 (PMC13700700; doi:10.3390/ani16203288)
Supplement: Supplementary file 1 [file animals-16-03288-s001.zip › Supplementary Materials_v03.pdf]

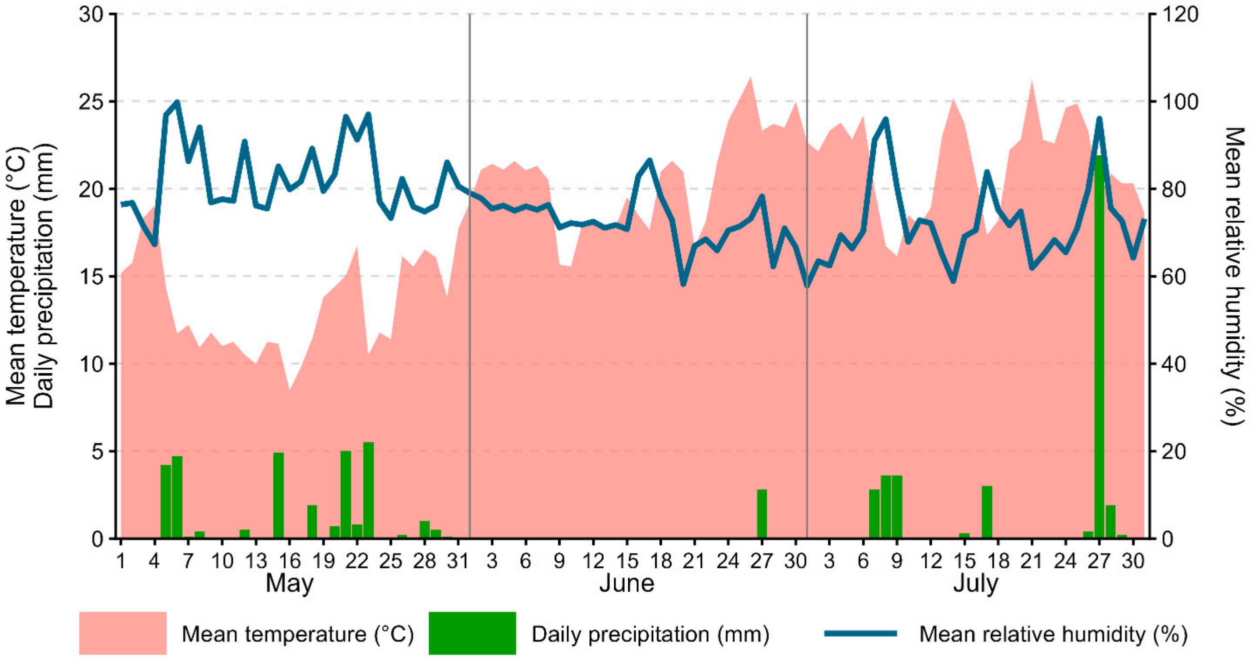

Figure S1. Daily weather conditions during the grazing period. Daily mean temperature, total daily precipitation, and mean relative humidity recorded from May to July 2025 by the on-site automated Boreas meteorological station.

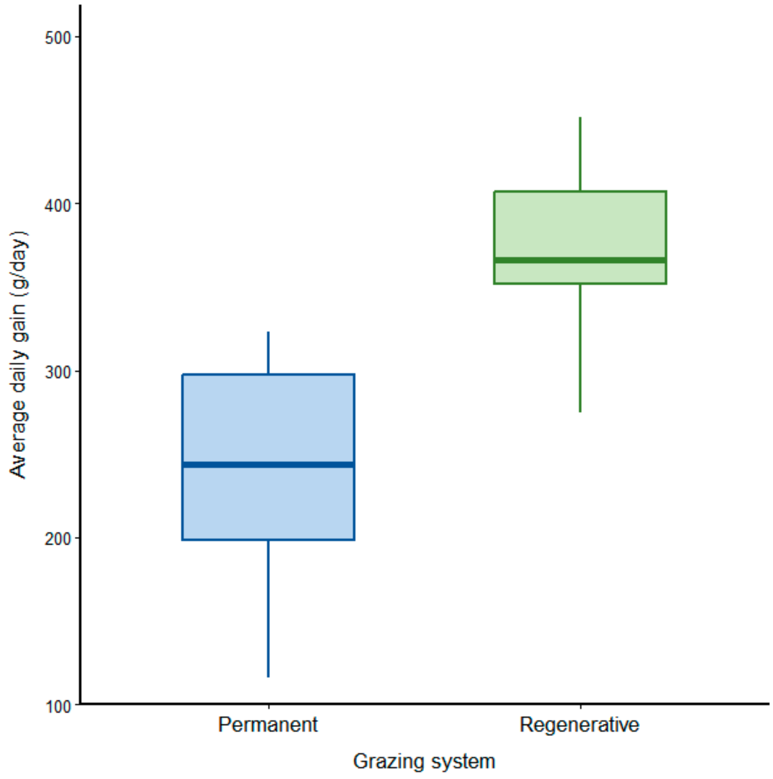

Figure S2. Average daily gain of donkeys under permanent and regenerative grazing systems. Boxes indicate interquartile ranges and horizontal lines represent median values (n = 12 per group).

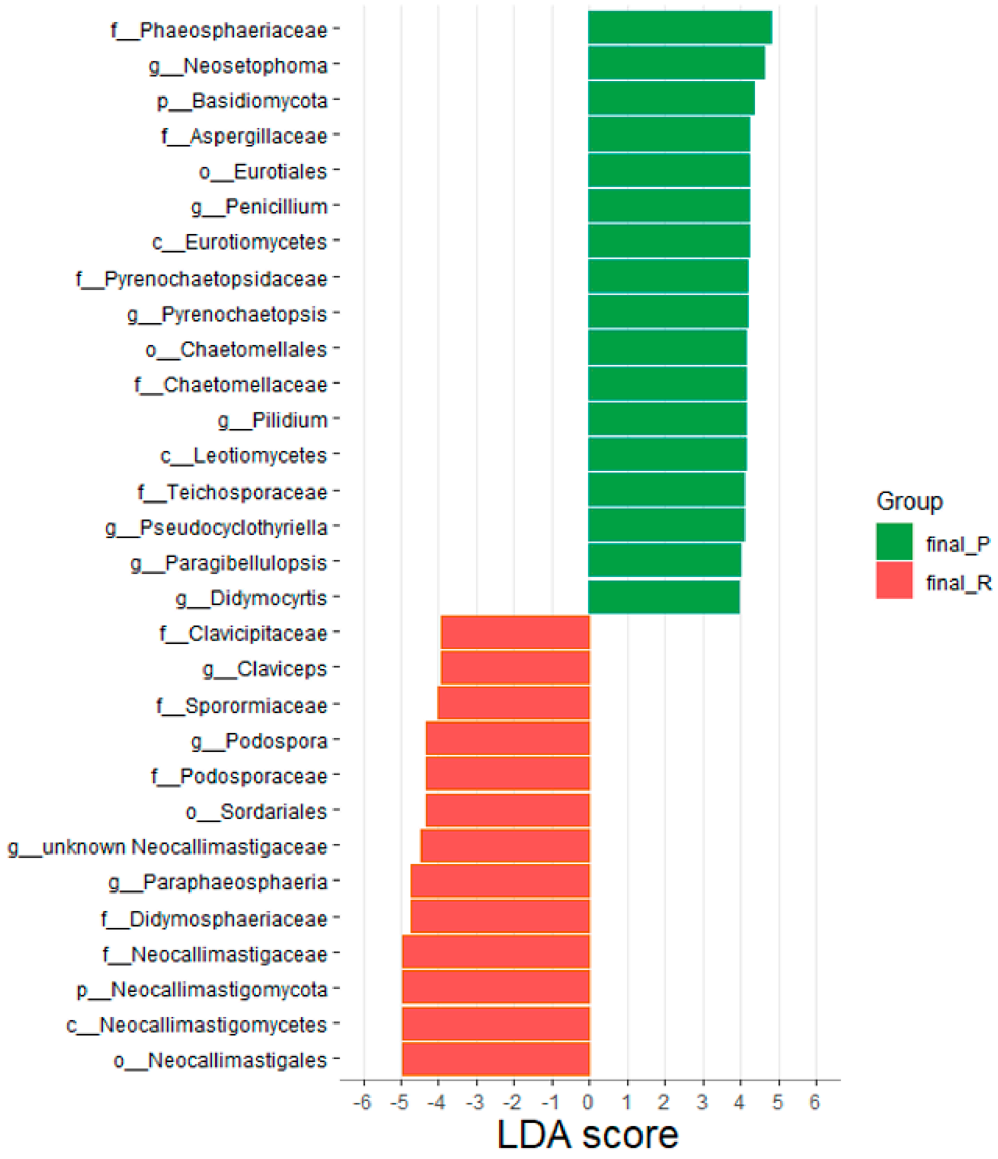

Figure S3. LEfSe analysis comparing fungal communities between final_P and final_R samples, highlighting end-point-specific fungal biomarkers. The top 30 discriminant fungal taxa are shown based on LDA scores. Taxa enriched in final_P are shown on the positive side of the axis, whereas taxa enriched in final_R are shown on the negative side. Taxonomic prefixes indicate phylum (p__), class (c__), order (o__), family (f__), and genus (g__). Group abbreviations: final_P, final samples from the permanent grazing system; final_R, final samples from the regenerative grazing system.

Table S1. Nutritional composition of grass hay provided to the animals prior to the grazing period.

Component	Value	Unit
**Moisture**	7.6	%
**Crude protein**	60	g/kg DM
**Crude fat**	14	g/kg DM
**Crude fiber**	296	g/kg DM
**Crude ash**	70	g/kg DM
**Neutral detergent fiber (NDF)**	537	g/kg DM
**Acid detergent fiber (ADF)**	347	g/kg DM
**Acid detergent lignin (ADL)**	62	g/kg DM

Values represent the chemical composition of grass hay fed to the animals during the winter feeding period preceding grazing.

Table S2. Stocking density and total live weight of animals in the two grazing systems

Parameter	Permanent grazing (5.3 ha)	Regenerative grazing (5.8 ha)
**Initial buffalo live weight (kg)**	2662.0	2686.5
**Initial donkey live weight (kg)**	1587.5	1560.5
**Total initial live weight (kg)**	4249.5	4247.0
**Livestock units per hectare**	1.60	1.46
**Initial instantaneous stocking density (kg live weight/ha)**	801.8	15377.1
**Final buffalo live weight (kg)**	3159.5	3256.5
**Final donkey live weight (kg)**	1826.0	1932.5
**Total final live weight (kg)**	4985.5	5189.0
**Livestock units per hectare**	1.88	1.79
**Final instantaneous stocking density (kg live weight/ha)**	940.7	18787.8

One livestock unit corresponds to 500 kg of live weight. Livestock units per hectare were calculated using the total pasture area. For the regenerative grazing system, stocking density expressed as kg live weight/ha was calculated as instantaneous stocking density for the currently grazed paddock, based on 21 paddocks of approximately equal size.

Table S3. Individual body weight gain and average daily gain of donkeys under regenerative and permanent grazing systems

Animal ID	Microbiome subset	Age (months)	Start BW (kg)	Final BW (kg)	Gain (kg)	ADG (g/day)	Grazing system
D34	Yes	12	154.5	177.0	22.5	274.39	Regenerative
D39	No*	11	137.0	166.0	29.0	353.66	Regenerative
D40	Yes	10	102.0	129.0	27.0	329.27	Regenerative
D41	Yes	12	117.0	150.0	33.0	402.44	Regenerative
D44	Yes	10	102.0	130.5	28.5	347.56	Regenerative
D45	Yes	12	152.0	182.0	30.0	365.85	Regenerative
D47	No*	12	136.5	165.5	29.0	353.66	Regenerative
D48	Yes	11	148.5	189.0	40.5	493.90	Regenerative
D51	No*	12	119.5	154.0	34.5	420.73	Regenerative
D56	Yes	11	165.0	195.0	30.0	365.85	Regenerative
D57	Yes	10	108.5	145.5	37.0	451.22	Regenerative
D58	No*	10	118.0	149.0	31.0	378.05	Regenerative
D35	Yes	11	152.5	167.0	14.5	176.83	Permanent
D36	Yes	11	122.0	148.0	26.0	317.07	Permanent
D37	No*	11	106.5	123.0	16.5	201.22	Permanent
D43	Yes	11	141.0	166.5	25.5	310.98	Permanent
D46	Yes	11	134.0	157.0	23.0	280.49	Permanent
D50	No*	10	122.5	149.0	26.5	323.17	Permanent
D52	Yes	11	129.0	153.0	24.0	292.68	Permanent
D53	Yes	11	133.5	149.0	15.5	189.02	Permanent
D54	Yes	12	153.0	171.0	18.0	219.51	Permanent
D55	No*	12	128.0	150.0	22.0	268.29	Permanent
D59	Yes	11	133.5	151.0	17.5	213.41	Permanent
D60	No*	12	132.0	141.5	9.5	115.85	Permanent

BW, body weight; ADG, average daily gain. The interval between the two body weight measurements was 82 days, of which the animals spent 70 days on pasture.

* It was not possible to collect fecal samples at both time points for these animals. Therefore, only body weight gain was analyzed in these cases.

Table S4. 16S rRNA gene amplicon sequencing summary and taxonomic composition

Sample ID	Grazing	Time	Nb domain	Nb phylum	Nb class	Nb order	Nb family	Nb genus	Nb species	Nb asv	Nb sequences
D35	Permanent	start	2	19	32	55	88	186	212	2404	129883
D36	Permanent	start	2	19	31	53	84	181	206	2422	105064
D43	Permanent	start	2	18	31	54	87	183	208	2450	115709
D46	Permanent	start	2	19	30	53	84	182	206	2356	132542
D52	Permanent	start	2	17	30	53	85	181	207	2371	123947
D53	Permanent	start	2	19	33	55	88	187	212	2491	152098
D54	Permanent	start	2	18	31	53	88	184	210	2360	114765
D59	Permanent	start	2	19	31	55	86	182	210	2355	117752
D34	Regenerative	start	2	19	32	54	84	180	202	2128	108869
D40	Regenerative	start	2	17	30	52	84	181	208	2419	133343
D41	Regenerative	start	2	17	30	54	86	182	206	2429	126616
D44	Regenerative	start	2	18	30	54	87	184	209	2319	134304
D45	Regenerative	start	2	19	33	55	90	188	213	2552	135198
D48	Regenerative	start	2	20	33	54	86	177	202	2314	138677
D56	Regenerative	start	2	16	28	50	84	185	210	2343	114721
D57	Regenerative	start	2	18	29	49	80	178	204	2103	127846
D35F	Permanent	final	2	18	31	52	83	179	207	2467	176795
D36F	Permanent	final	2	19	29	50	82	178	201	2431	162760
D43F	Permanent	final	2	20	32	55	88	185	210	2527	165253
D46F	Permanent	final	2	17	27	49	81	170	193	2195	172060
D52F	Permanent	final	2	19	30	54	87	183	206	2352	187927
D53F	Permanent	final	2	20	34	57	90	188	215	2554	192241
D54F	Permanent	final	2	19	32	56	89	186	211	2451	185043
D59F	Permanent	final	2	18	31	53	84	182	207	2506	149886
D34F	Regenerative	final	2	18	32	56	89	187	214	2448	151782
D40F	Regenerative	final	2	18	31	55	88	186	212	2478	177653
D41F	Regenerative	final	2	18	31	53	86	186	213	2454	127081
D44F	Regenerative	final	2	17	28	52	85	183	208	2402	159740
D45F	Regenerative	final	2	19	33	55	89	187	214	2526	142538
D48F	Regenerative	final	2	19	33	55	86	184	209	2525	131305
D56F	Regenerative	final	2	19	33	53	87	187	213	2525	140132
D57F	Regenerative	final	2	19	31	54	87	186	210	2274	117313

Sample ID	Grazing	Time	Nb domain	Nb phylum	Nb class	Nb order	Nb family	Nb genus	Nb species	Nb asv	Nb sequences
**total[1]**	**-**	**-**	**2**	**20**	**34**	**59**	**94**	**195**	**223**	**2833**	**4550843**
start_P[2]	Permanent	start	2.00±0.00	18.50±0.76	31.13±0.99	53.88±0.99	86.25±1.75	183.25±2.25	208.88±2.47	2401.13±50.25	-
start_R[2]	Regenerative	start	2.00±0.00	18.00±1.31	30.63±1.85	52.75±2.19	85.13±2.90	181.88±3.68	206.75±3.96	2325.88±151.07	-
final_P[2]	Permanent	final	2.00±0.00	18.75±1.04	30.75±2.12	53.25±2.82	85.50±3.42	181.38±5.71	206.25±6.73	2435.38±115.54	-
final_R[2]	Regenerative	final	2.00±0.00	18.38±0.74	31.50±1.69	54.13±1.36	87.13±1.46	185.75±1.49	211.63±2.33	2454.00±85.34	-

[1]: In the “total” row, sequencing depth represents the sum of reads across samples, whereas taxonomic relative abundances and ASV counts are calculated from the pooled dataset rather than summed across samples.

[2]: Group-level mean values ± standard deviation are provided for descriptive purposes only; statistical comparisons were performed using dedicated diversity and differential abundance analyses.

Table S5. ITS gene amplicon sequencing summary and taxonomic composition

Sample ID	Grazing	Time	Nb domain	Nb phylum	Nb class	Nb order	Nb family	Nb genus	Nb species	Nb asv	Nb sequences
D35	Permanent	start	1	3	12	22	40	67	98	241	27984
D36	Permanent	start	1	3	11	22	39	68	96	256	37485
D43	Permanent	start	1	4	13	21	38	69	99	259	39893
D46	Permanent	start	1	3	12	23	43	82	124	302	81606
D52	Permanent	start	1	3	13	25	45	75	105	256	60459
D53	Permanent	start	1	4	14	25	41	68	99	270	71117
D54	Permanent	start	1	6	17	29	55	105	167	355	62750
D59	Permanent	start	1	4	15	23	46	89	134	317	111275
D34	Regenerative	start	1	4	12	19	34	63	90	251	26760
D40	Regenerative	start	1	4	13	23	40	70	100	269	56036
D41	Regenerative	start	1	4	13	21	36	71	102	267	88414
D44	Regenerative	start	1	4	12	23	42	71	103	272	66088
D45	Regenerative	start	1	4	13	23	42	77	121	303	62325
D48	Regenerative	start	1	4	14	25	50	82	117	284	48919
D56	Regenerative	start	1	4	14	29	54	96	144	305	61711
D57	Regenerative	start	1	5	17	28	56	104	162	356	90506
D35F	Permanent	final	1	4	15	32	73	126	189	385	68964
D36F	Permanent	final	1	6	18	35	79	137	201	427	121744
D43F	Permanent	final	1	4	16	35	82	135	205	434	100926
D46F	Permanent	final	1	4	16	34	75	128	201	339	131225
D52F	Permanent	final	1	5	18	36	76	127	194	381	92250
D53F	Permanent	final	1	5	17	36	77	129	193	398	138553
D54F	Permanent	final	1	4	16	32	69	126	185	389	102090
D59F	Permanent	final	1	5	17	34	76	131	196	395	136365
D34F	Regenerative	final	1	5	18	34	75	122	180	391	60847
D40F	Regenerative	final	1	5	18	33	67	102	148	341	59171
D41F	Regenerative	final	1	3	17	31	72	118	171	352	58452
D44F	Regenerative	final	1	3	15	32	73	117	174	344	76168
D45F	Regenerative	final	1	3	16	29	59	91	124	320	63530
D48F	Regenerative	final	1	4	14	30	58	90	124	291	15284
D56F	Regenerative	final	1	5	16	30	68	105	150	342	100054
D57F	Regenerative	final	1	5	16	29	62	95	142	316	35037

Sample ID	Grazing	Time	Nb domain	Nb phylum	Nb class	Nb order	Nb family	Nb genus	Nb species	Nb asv	Nb sequences
**total[1]**	**-**	**-**	**1**	**6**	**21**	**40**	**92**	**172**	**282**	**596**	**2353988**
start_P[2]	Permanent	start	1.00±0.00	3.75±1.04	13.38±1.92	23.75±2.55	43.38±5.48	77.88±5.48	115.25±25.09	282.00±39.02	-
start_R[2]	Regenerative	start	1.00±0.00	4.13±0.35	13.50±1.60	23.88±3.36	44.25±8.17	79.25±14.10	117.38±24.52	288.38±32.91	-
final_P[2]	Permanent	final	1.00±0.00	4.63±0.74	16.63±1.06	34.25±1.58	75.88±3.87	129.88±4.16	195.50±6.68	393.50±29.28	-
final_R[2]	Regenerative	final	1.00±0.00	4.13±0.99	16.25±1.39	31.00±1.85	66.75±6.50	105.00±12.71	151.63±21.76	337.13±29.46	-

[1]: In the "total" row, sequencing depth represents the sum of reads across samples, whereas taxonomic relative abundances and ASV counts are calculated from the pooled dataset rather than summed across samples.

[2]: Group-level mean values ± standard deviation are provided for descriptive purposes only; statistical comparisons were performed using dedicated diversity and differential abundance analyses.

Table S6. Summary of longitudinal DESeq2 differential-abundance analysis based on the grazing system × sampling time interaction

Dataset	ASVs analyzed	ASVs with valid adjusted p-value	Significant interaction ASVs	Positive interaction	Negative interaction	Significant ASVs (%)
16S	2833	2833	336	195	141	11.86
ITS	596	480	66	41	25	11.07

Positive interaction indicates a more positive temporal change from start to final under regenerative grazing relative to permanent grazing; negative interaction indicates the opposite direction. Significance was defined as Benjamini-Hochberg-adjusted $p < 0.05$.

Detailed ASV-level results, including taxonomic assignments, interaction log2 fold changes, raw *p*-values, adjusted *p*-values, and additional within-group statistics, are provided in Table S7.
